# Ultrasound-activated phase-change nanodroplets for root canal biofilm eradication: mechanistic Investigation and experimental validation

**DOI:** 10.3389/fbioe.2026.1882780

**Published:** 2026-10-28

**Authors:** Xiaoyu Qian, Jie Dang, Ziqi Shi, Jue Zhang

**Affiliations:** 1 Academy for Advanced Interdisciplinary Studies, Peking University, Beijing, China; 2 School of Mechanics and Engineering Science, Peking University, Beijing, China

**Keywords:** acoustic cavitation dynamics, eradication efficiency validation, phase transition simulation, phase-change nanodroplets, root canal biofilm, ultrasound activation

## Abstract

Refractory root canal infections pose a major clinical challenge, as conventional treatments fail to eradicate biofilms in the complex narrow root canal structure. This study proposed a novel strategy of ultrasound-activated phase-change nanodroplets (PCNDs) for biofilm elimination and elucidated its physicochemical and biomechanical mechanisms. A coupled Yang–Church–Pitt (YCP) dynamic model was established to simulate the complete phase transition-cavitation-energy release process of PCNDs under ultrasonic irradiation. *Ex vivo* experiments on *E. faecalis* biofilms confirmed that PCNDs efficiently disrupted biofilms via cavitation effects, with efficacy positively correlated with ultrasonic intensity but no significant frequency influence. Minimal temperature of approximately 2 °C rises during treatment verified the strategy’s biosafety, preventing dental hard tissue damage. The YCP model accurately predicted the system’s cavitation characteristics and thermal dynamics, and simulated cavitation energy was strongly correlated with experimental biofilm elimination efficiency. This work validated the safety and efficacy of ultrasound-activated PCNDs for root canal biofilm removal; the YCP model provided a theoretical framework for optimizing ultrasonic parameters, offering a novel mechanistically informed approach for managing refractory root canal infections.

## 1 Introduction

Refractory root canal diseases remain difficult to treat, largely due to the failure of traditional therapies to clear biofilms from the narrow and complex root canal structure (Boutsioukis and Arias-Moliz, 2022). The root canal system features intricate anatomical characteristics, such as dentinal tubules with diameters ranging from 2 to 20 μm, which serve as ideal niches for bacterial colonization and biofilm formation (Tonini et al., 2022). Root canal infections are typically polymicrobial, involving complex multispecies biofilms with substantial interindividual variation in microbial composition (Pinto et al., 2023; Siqueira and Rôças, 2009). Nevertheless, *E. faecalis* is frequently associated with persistent and secondary endodontic infections and was therefore selected as a representative single-species model in this study because of its strong biofilm-forming capacity and tolerance to adverse intracanal conditions (Stuart et al., 2006; Yang et al., 2024). Biofilms produce a dense extracellular matrix composed of adhesive proteins, polysaccharides, and lipids (Koo et al., 2017). This matrix acts as a physical and chemical barrier, reducing the penetration efficiency of antibiotics by 100–1000 times compared to their effect on planktonic bacteria (Flemming et al., 2016). Moreover, bacteria within biofilms often enter a dormant state with reduced metabolic activity, further enhancing their resistance to antimicrobial agents, making complete eradication extremely challenging.

Conventional strategies for biofilm removal mainly rely on mechanical instrumentation combined with chemical irrigation using sodium hypochlorite, EDTA, and chlorhexidine, as well as irrigation activation techniques (Boutsioukis and Arias-Moliz, 2022; Nagendrababu et al., 2018). However, their effectiveness is often limited by the complex root canal anatomy and the protective extracellular polymeric matrix of mature biofilms (Kumar et al., 2023; Sen et al., 2024). In recent years, phase-change nanodroplets (PCNDs) have emerged as promising candidates for addressing biofilm-related infections, owing to their ability to mechanically disrupt biofilms and enhance antimicrobial penetration under ultrasound activation (Kripfgans et al., 2000; Loskutova et al., 2019; Frinking et al., 2020). Under ultrasonic stimulation, PCNDs undergo a liquid-to-gas phase transition, generating microbubbles that induce cavitation effects (Sheeran and Dayton, 2012; Sheeran et al., 2012; Shpak et al., 2014). This cavitation process—characterized by the formation, growth, and violent collapse of microbubbles—releases substantial mechanical energy that can physically disrupt biofilm structures (Lattwein et al., 2020; Durham et al., 2021). Cavitation activity has been extensively characterized using established passive and active acoustic methods (Chowdhury et al., 2020). Passive detection identifies the nonlinear emissions of oscillating bubbles, whereas passive cavitation imaging and active ultrafast imaging can further provide spatial and temporal information on cavitation events (Lattwein et al., 2022; Haworth et al., 2017). These approaches have been widely applied to characterize microbubble activity and acoustic droplet vaporization, establishing a well-developed experimental framework for relating acoustic emissions to bubble dynamics and therapeutic bioeffects (Gyöngy and Coussios, 2010; Gateau et al., 2011). Unlike traditional microbubbles (with diameters of 1–10 μm) that fail to penetrate narrow dentinal tubules or reach apical regions, PCNDs (with diameters typically <500 nm) exhibit superior penetration capabilities, making them well-suited for root canal applications (Loskutova et al., 2019; Frinking et al., 2020).

The study of acoustic cavitation dates back over a century, with numerous theoretical models developed to describe bubble dynamics (Yasui, 2018). Early models, such as the Rayleigh-Plesset equation, laid the foundation for understanding bubble collapse in Newtonian fluids but failed to account for the viscoelastic properties of biological tissues (Rayleigh, 1917). Subsequent models, including the Fogler-Goddard equation, which accounts for viscous and elastic effects of the surrounding medium, and the Yang-Church equation, which further describes bubble dynamics in viscoelastic soft tissues, incorporated viscoelasticity to better simulate bubble behavior in biological tissues (Fogler and Goddard, 1970; Yang and Church, 2005). However, these models primarily focus on microbubble vibration and do not consider the phase transition process of nanodroplets, limiting their applicability to PCND-based systems (Yasui, 2018).

The Pitt model addressed this gap by exploring the mathematical laws of acoustically induced droplet phase transition, providing a framework for calculating heat transfer during the liquid-to-gas conversion of perfluorocarbon (PFC) nanodroplets (Sheeran et al., 2012). While the Pitt model accurately describes phase transition thermodynamics, it lacks a detailed account of microbubble cavitation dynamics and energy release, which are critical for explaining biofilm destruction (Kripfgans et al., 2000). Thus, a comprehensive model that integrates phase transition thermodynamics and cavitation dynamics is urgently needed to bridge the gap between theoretical predictions and experimental observations.

To overcome the limitations of existing models, the present study integrates the Yang-Church equation (describing cavitation bubble vibration in viscoelastic media) with the Pitt model (describing phase transition heat transfer of nanodroplets) to establish a novel YCP dynamic model (Loskutova et al., 2019; Sheeran et al., 2012). This integration enables the simulation of the entire “phase transition-cavitation-energy release” process of PFC nanodroplets under ultrasound. Specifically, the Yang-Church equation is used to quantify the vibration behavior of microbubbles formed after phase transition, considering the viscoelasticity of dental tissues (Pitt et al., 2014). The Pitt model is employed to calculate heat transfer during the phase transition of perfluoropentane (PFP, a member of the PFC family) nanodroplets, including heat conduction at the liquid-gas interface and latent heat of vaporization (Sheeran et al., 2012). By coupling these two models, the YCP model can predict key parameters such as microbubble maximum vibration radius, cavitation energy release, and system temperature changes—all of which are critical for understanding biofilm destruction mechanisms (Fan et al., 2021; Doinikov, 1999).

Although single-bubble models have been widely used to investigate cavitation dynamics because of their computational efficiency and clear physical interpretation, increasing evidence indicates that interactions among neighboring bubbles may modify oscillation amplitude, collapse behavior, acoustic emission, and energy transfer through secondary radiated pressure, multiple scattering, and secondary Bjerknes forces (Barbat et al., 1999; Reddy and Szeri, 2002; Allen et al., 2003). Consequently, coupled multi-bubble and bubble-cloud models have become an important direction in cavitation research (Fuster and Colonius, 2011). Nevertheless, these approaches generally require detailed knowledge of bubble number density, spatial distribution, activation fraction, and surrounding acoustic conditions, which are difficult to determine experimentally. Therefore, the present study employs a representative-bubble Yang–Church–Pitt (YCP) model to investigate the local dynamic response of vaporized PCNDs (Tiwari et al., 2015; Ohl et al., 2015).

We hypothesized that ultrasound-activated PFP-based PCNDs could efficiently disrupt root canal biofilms through cavitation effects, and that the YCP model could accurately predict the cavitation dynamics under different ultrasonic and material conditions.

The primary objectives of this study are: 1. to fabricate stable PFP-based PCNDs and validate their ability to eliminate root canal biofilms under ultrasound activation; 2. to use the YCP model to simulate the dynamic process of ultrasound-activated PCNDs and analyze the effects of ultrasonic parameters (frequency, intensity) and material properties (surface tension, shear modulus) on cavitation efficiency; 3. to verify the validity of the YCP model by comparing simulation results with experimental data (biofilm destruction rate, temperature changes).

Experimental work focuses on *E. faecalis* biofilms (7-day old) grown on *ex vivo* human teeth, simulating early and refractory root canal infections, respectively. Ultrasonic parameters are varied (frequency: 1–3 MHz; intensity: 1–3 W/cm^2^) to evaluate their impact on biofilm elimination. The YCP model is used to simulate these processes, with results validated against multi-modal microscopy and temperature measurement data.

## 2 Materials and methods

### 2.1 YCP theory

#### 2.1.1 Overview of the simulation model

Under ultrasonic irradiation, when the acoustic pressure exceeds the PFP phase transition threshold, the liquid PFP core rapidly undergoes liquid-to-gas vaporization with a volume expansion, forming microbubbles (Kripfgans et al., 2000). Sustained ultrasonic driving induces nonlinear vibration of these microbubbles, which expand to a maximum radius before undergoing violent collapse (acoustic cavitation) (Sheeran et al., 2012). This expansion-collapse process releases intense shockwaves and microjets, the core physical effects for biofilm disruption (Shpak et al., 2014).

The YCP model is developed to describe the coupled processes of phase transition of PFP nanodroplets and cavitation of the resulting microbubbles. It builds on the Yang-Church model for bubble dynamics in viscoelastic media and the Pitt model for phase transition heat transfer, integrating them to capture the multi-physical interactions between ultrasound, nanodroplets, and biofilms (Chowdhury et al., 2020; Ohl et al., 2006). Default parameters and values and more detailed derivation are provided in the Supplementary Material. The overall modeling framework and the sequential phase-transition and cavitation processes are illustrated in Figures 1, 2, respectively.

#### 2.1.2 Microbubble dynamics based on the Yang-Church (YC) model

The YC model describes the nonlinear vibration of cavitation bubbles in viscoelastic media, accounting for inertial forces, surface tension, viscous damping, and ultra-sonic driving pressure (Yang and Church, 2005). Two key conservation laws form the basis of the model:

The mass conservation equation considers the constant composition of the bubble membrane and the effect of temperature-dependent bubble density on phase transition. For a spherical bubble, the equation is derived as:

$$\rho_{g}4\pi R^{2}\frac{dR}{dt}-\frac{4}{3}\pi \left(R^{3}-R_{L}^{3}\right)\left(\frac{p_{g}\bar{M_{w}}}{\Gamma {\bar{T_{g}}}^{2}}\right)\frac{d\bar{T_{g}}}{dt}=\left(\bar{M_{w}}-\frac{\rho_{g}}{\rho_{PFP}}X_{PFP}M_{PFP}\right)\frac{dN}{dt}$$ (1)

where R is the instantaneous bubble radius, $R_{L}$ is the initial nanodroplet radius, $\rho_{g}$ is the gas density inside the bubble, $p_{g}$ is the gas pressure, $\bar{M_{w}}$ is the average molecular weight of the gas mixture, Γ is the ideal gas constant, $\bar{T_{g}}$ is the average temperature of the bubble membrane, $X_{PFP}$ is the mole fraction of PFP in the gas phase, $M_{PFP}$ is the molar mass of PFP, and N is the total moles of gas. This equation quantifies the mass exchange between the liquid nanodroplet and the gaseous bubble during phase transition.

The energy conservation equation for microbubble vibration in viscoelastic media is:

$$\left(1-\frac{v_{R}}{c}\right)Ra_{R}+\frac{3}{2}\left(1-\frac{v_{R}}{3c}\right)v_{R}^{2}=\left(1+\frac{v_{R}}{c}\right)\frac{p_{\Delta }}{\rho }+\frac{Rd\left(p_{\Delta }\right)}{\rho cdt}$$ (2)

here where $v_{R}$ is the bubble wall velocity, $a_{R}$ is the bubble wall acceleration, c is the speed of sound in the medium, $p_{\Delta }$ is the pressure difference across the bubble wall, and ρ is the medium density. This equation describes the conversion of ultrasonic energy into mechanical energy during bubble vibration, which drives biofilm disruption.

#### 2.1.3 Phase transition heat transfer based on the Pitt model

The Pitt model focuses on the heat transfer processes during the acoustic droplet vaporization (ADV) of PFP nanodroplets, considering heat conduction, latent heat of vaporization, and thermal radiation (negligible in this system) (Sheeran and Dayton, 2012; Pitt et al., 2014). The key energy balance equation at the liquid-gas interface is given in Equation 3:

$$q_{s,PFP}=q_{c,gas}+q_{rad}+q_{phasechange}$$ (3)

where $q_{s,PFP}$ is the total heat flux at the PFP droplet surface, $q_{c,gas}$ is the heat conduction flux from the surrounding medium to the droplet, and $q_{phasechange}$ is the heat flux consumed by vaporization.

The Pitt model also accounts for temperature variations during phase transition, which affect the stability and cavitation behavior of the resulting microbubbles. The temperature change of the PFP droplet is calculated as:

$$\frac{dT_{droplet}}{dt}=\frac{3}{\rho C_{m,PFP}R_{L}}\left[\theta \frac{k_{gas}}{R_{L}}\left(T_{s,aq}-T_{s,PFP}\right)-\frac{X_{PFP}M_{PFP}{\Delta H}_{PFP}}{4\pi R_{L}^{2}}\frac{dN}{dt}\right]$$ (4)

where $T_{droplet}$ is the droplet temperature, $C_{m,PFP}$ is the specific heat capacity of PFP, $k_{gas}$ is the thermal conductivity of the gas, $T_{s,aq}$ is the temperature of the surrounding aqueous medium, $T_{s,PFP}$ is the temperature of the PFP-liquid interface, and ${\Delta H}_{PFP}$ is the latent heat of vaporization of PFP (Sheeran et al., 2012). This equation captures the cooling effect of PFP vaporization, which influences the viscoelastic properties of the surrounding medium and, in turn, bubble cavitation.

#### 2.1.4 Solution of the YCP model and calculation of simulation metrics

Combined with the Bernoulli equation and boundary conditions for viscoelastic media, the Pitt model is extended and coupled with the Yang-Church cavitation bubble dynamics model to establish the integrated YCP model. Unlike traditional single models that only describe either cavitation or phase transition, this coupled framework simultaneously accounts for both viscoelastic cavitation behavior and phase transition thermodynamics, enabling more accurate prediction of microbubble vibration characteristics and energy release during ultrasonic activation (Sheeran et al., 2012).

The YCP model is governed by three coupled differential equations (Equations 1, 2, 4), which form a closed system with three core unknown variables that fully characterize the dynamic evolution of PCNDs under ultrasonic irradiation: the microbubble radius R (describing the expansion and collapse dynamics of vaporized microbubbles), the molar amount of PFP undergoing phase transition per unit time $\frac{dN}{dt}$ (quantifying the liquid-to-gas vaporization kinetics of the PFP liquid core), and the PFP-liquid interface temperature $T_{s,PFP}$ (reflecting heat exchange during phase transition and the system’s temperature-dependent thermodynamic behavior). This closed system of three equations with three unknowns can be solved numerically using the ODE45 solver (an adaptive Dormand–Prince Runge–Kutta solver) in MATLAB, which performs differential equation integration to obtain the full dynamic evolution process of PCNDs from phase transition to cavitation collapse, including time-varying curves of microbubble radius, phase transition rate, and system temperature.

To evaluate cavitation efficiency and biofilm destruction potential, four key metrics are calculated from the YCP model:

- Cavitation energy ( $W_{therm}$ ):

$$W_{therm}=p_{0}V_{0}\left(\frac{1-{\left(\frac{n_{2}T_{2}R_{1}^{3}}{n_{1}T_{1}R_{2}^{3}}\right)}^{\frac{\gamma -1}{\gamma }}}{\gamma -1}-\ln\left(\frac{n_{1}T_{1}R_{0}^{3}}{n_{0}T_{0}R_{1}^{3}}\right)\right)$$ (5)

The cavitation energy Wtherm in Equation 5 is computed by dividing each cavitation process into two stages: with the total work and heat dissipation throughout the process calculated as $W_{therm}$ , where subscripts 0, 1, and 2 represent the initial state of the droplet, the maximum expansion state of the microbubble, and the post-collapse contraction state, respectively (Lattwein et al., 2022);

- Quench time (*t*): the quench time t is defined as the time interval between the first maximum expansion and first minimum contraction of the microbubble, calculated as $t=t_{minlocal}-t_{maxlocal}$ ;
- Global expansion ratio (*rationglo*): the global expansion ratio *ratioglo* is calculated from the maximum and minimum microbubble radii obtained from the full numerical simulation, defined as $ratioglo=R_{\max}/R_{\min}$ ;
- Local expansion ratio (*rationlocal*): the local expansion ratio *rationlocal*​ is derived from the first maximum and minimum radii in the initial vibration cycle, given by $rationlocal=R_{maxlocal}/R_{minlocal}$ .

Among these metrics, cavitation energy $W_{therm}$ ​ is the most critical, serving as an approximate calculation of the mechanical energy released during cavitation. Both expansion ratios describe the expansion-contraction behavior of microbubbles during simulation and are correlated with cavitation energy. It should be emphasized that cavitation energy is not maximized for optimal performance; excessively high energy poses risks to dental hard tissue and the oral physiological environment. Therefore, all four evaluation metrics must be controlled within an optimal range to ensure both therapeutic efficacy and biological safety, providing a comprehensive theoretical basis for the optimization of ultrasonic treatment parameters and material properties (Yang and Church, 2005).

The original YCP formulation was used as a reduced-order model of a representative vaporized PCND. To assess the sensitivity of its predictions to neighboring-bubble interaction, an additional reciprocally coupled two-bubble analysis was performed. The secondary acoustic pressure radiated by each bubble was introduced as an additional pressure term in the radial-dynamic equation of the neighboring bubble, while the original phase-transition, heat-transfer, and viscoelastic constitutive components were retained. The full equations, parameter sources, numerical implementation, validity criteria, and results are provided in Supplementary Section 4.

### 2.2 Experimental materials

Perfluoropentane (purity ≥99%) was used as the liquid core of phase-change nanodroplets (PCNDs), and detailed information of fabrication of PCNDs is provided in Supplementary Material Section S3 (Vlatakis et al., 2024). Human teeth used in this study were clinically collected at Peking University School and Hospital of Stomatology, Beijing, China, following clinical dental treatment. The teeth were obtained after clinical extraction and were not extracted specifically for the purpose of this research. Accordingly, the extraction was undertaken for clinical indications rather than to obtain research specimens. Adult single-rooted anterior teeth with fully developed apices and without caries, cracks, or external root resorption were selected. The teeth were cleaned to remove calculus and residual periodontal tissues and were subsequently prepared as *ex vivo* specimens for the root canal biofilm model. The processed teeth were sterilized by autoclaving at 121 °C for 15 min before use. Human tooth sample collection and all experimental procedures involving the human teeth were conducted in accordance with protocols approved by the Research Ethics Committees of Peking University School and Hospital of Stomatology. *Enterococcus faecalis* (ATCC 29212) was used as the model pathogen for root canal biofilm, cultured in brain heart infusion (BHI) broth (supplemented with 5% fetal bovine serum) at 37 °C under 5% CO_2_ (Lin et al., 2021).

Ultrasonic treatment was performed using an ultrasonic therapeutic apparatus (UT1041, Sonic-Stimu Pro, Shenzhen Dongdixin Technology Co., Ltd., Shenzhen, China). Ultrasound was applied at frequencies of 1–3 MHz with intensities of 1–3 W/cm^2^ and a duty cycle of 50%, with a flat transducer (diameter: 10 mm) positioned perpendicular to the sample surface at a fixed distance of 5 mm. Additional technical specifications are provided in Supplementary Section S3.2 (Durham et al., 2021).Temperature changes during treatment were monitored using an infrared thermography camera (resolution: 0.05 °C; frame rate: 30 fps), and biofilm characterization was conducted using a scanning electron microscope (SEM, acceleration voltage: 10 kV) and a confocal laser scanning microscope (CLSM, excitation wavelength: 488 nm for live bacteria, 561 nm for dead bacteria).

### 2.3 Experimental design

#### 2.3.1 Ultrasound parameter setup

To evaluate the effects of ultrasonic frequency and intensity on biofilm elimination, experiments were designed with two frequency levels (1 MHz and 3 MHz, covering the clinically commonly used ultrasonic endodontic therapy range) and three intensity levels (1 W/cm^2^, 2 W/cm^2^, 3 W/cm^2^, within the safety threshold for dental hard tissue to avoid thermal damage). A total of 4 groups were set for each frequency (n = 6 per group based on previous studies) to ensure statistical robustness:

- Group A (Blank Control): Samples treated with phosphate-buffered saline (PBS) only, no ultrasound or PCNDs;
- Group B (Low-Intensity Ultrasound): Samples treated with PCNDs + ultrasound at 1 W/cm^2^;
- Group C (Medium-Intensity Ultrasound): Samples treated with PCNDs + ultrasound at 2 W/cm^2^;
- Group D (High-Intensity Ultrasound): Samples treated with PCNDs + ultrasound at 3 W/cm^2^.

All ultrasonic treatments were conducted for a fixed duration of 5 min, consistent with the typical clinical ultrasonic irrigation time, to ensure comparability between groups.

#### 2.3.2 Biofilm and PCNDs preparation

Briefly, sterilized tooth samples were immersed in *E. faecalis* suspension (1 × 10^8^ CFU/mL) and cultured at 37 °C under 5% CO_2_ for 7 days to form mature biofilms (simulating early root canal infections). The culture medium was refreshed every 48 h to maintain nutrient supply and stable bacterial growth. Before ultrasonic treatment, samples were gently rinsed with PBS to remove planktonic bacteria, ensuring only adherent biofilms were tested.

PFP-based PCNDs were prepared using a modified thin-film hydration and ultrasonic emulsification method. The nanodroplets consisted of a PFP liquid core stabilized by a composite interfacial layer containing DSPC, DSPE-PEG-2K, and Pluronic. After emulsification, washing, and resuspension, the PCND concentration was adjusted to 1.0 × 10^9^ particles/mL and stored at 4 °C before use. Particle size distribution, zeta potential, morphology, and storage stability were evaluated using dynamic light scattering and transmission electron microscopy. Detailed preparation and characterization procedures are provided in Supplementary Section S3.1.

#### 2.3.3 Temperature measurement experiment

To verify the temperature prediction of the YCP model, temperature changes in the system (including the PCND suspension and tooth sample) were monitored in real time during ultrasonic treatment. The infrared thermography camera was calibrated using a standard temperature probe (accuracy: ±0.1 °C) before the experiment. Temperature data were recorded at 1-min intervals from the start of ultrasound irradiation to 5 min after treatment (to capture both the heating phase during ultrasound and the cooling phase after cessation). A blank control group (PCND suspension without ultrasound) was also set to exclude temperature changes caused by environmental factors (Lau et al., 2023).

#### 2.3.4 Biofilm elimination efficacy assessment

After ultrasonic treatment, biofilm elimination efficacy was evaluated quantitatively using three complementary methods: SEM Observation, CLSM Analysis. Detailed assessment protocols are provided in the Supplementary Material due to their technical complexity.

All experiments were repeated three times independently to ensure result reproducibility, and statistical analysis was performed using one-way analysis of variance (ANOVA) with Tukey’s post-hoc test (significance level: P < 0.05).

## 3 Results

### 3.1 Simulation results from the YCP model

The YCP model was established to simulate the entire “phase transition-cavitation-energy release” process of PFP nanodroplets under ultrasound irradiation. A comprehensive set of parameters was incorporated into the simulation, covering two core categories: material properties and ultrasonic parameters. Material property parameters included surface tension *σ* (0.04–0.1 N/m), shear modulus *G* (0–2 MPa), initial particle size *R*_*0*_ (1 × 10^−7^∼4 × 10^−7^ m), *μ* viscosity (0–0.01 Pa·s), sound speed *c* (340–1500 m/s), and density *ρ* (500–2000 kg/m^3^). Ultrasonic parameters focused on sound pressure *p*_1_ (1.73 × 10^5^–9 × 10^5^ Pa) and frequency *f*_1_ (0.5 × 10^6^–2 × 10^6^ Hz). The simulation aimed to clarify the effects of these parameters on key cavitation metrics, including cavitation energy ( $W_{therm}$ ​), quenching time (*t*), global expansion ratio (*rationglo*), and local expansion ratio (*rationlocal*), providing a theoretical basis for optimizing treatment parameters (Loskutova et al., 2019; Sheeran et al., 2012).

#### 3.1.1 Effects of ultrasonic parameters on cavitation behavior

Ultrasonic parameters, particularly sound pressure and frequency, were identified as critical factors directly regulating the cavitation behavior of PCNDs. Figure 3 was employed to characterize the trend of bubble vibration under different ultrasonic frequencies, focusing on qualitative changes rather than specific quantitative values. The results clearly showed that frequency primarily affects the cycle and speed of bubble vibration rather than the total cavitation energy released. At a fixed sound pressure, as the ultrasonic frequency increased, the bubble vibration cycle shortened significantly, and the quenching time (the duration from bubble maximum expansion to collapse) decreased accordingly. However, across the entire tested frequency range of 0.5–2 MHz, the cavitation energy remained relatively stable, with values fluctuating only slightly between 8.49 × 10^−14^ J and 8.52 × 10^−14^ J, confirming that frequency mainly modulates the dynamic process of cavitation rather than the total energy output.

**FIGURE 1:**
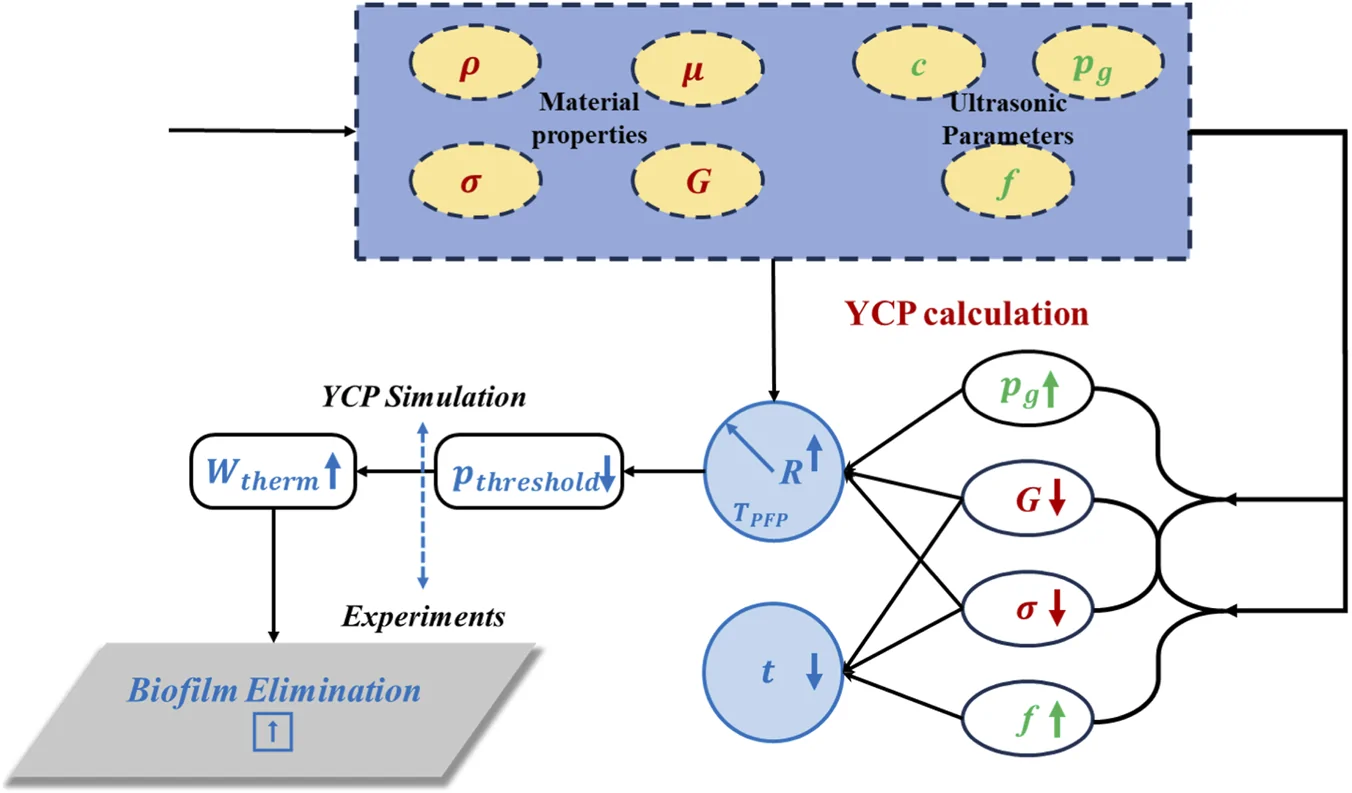
Schematic diagram of the YCP dynamic model framework and its experimental validation. Input material and ultrasonic parameters are used for both model simulation and *ex vivo* experiments. The model predicts microbubble cavitation dynamics and temperature changes, which are validated by experimental biofilm elimination and temperature monitoring, confirming the model’s predictive power for clinical parameter optimization.

**FIGURE 2:**
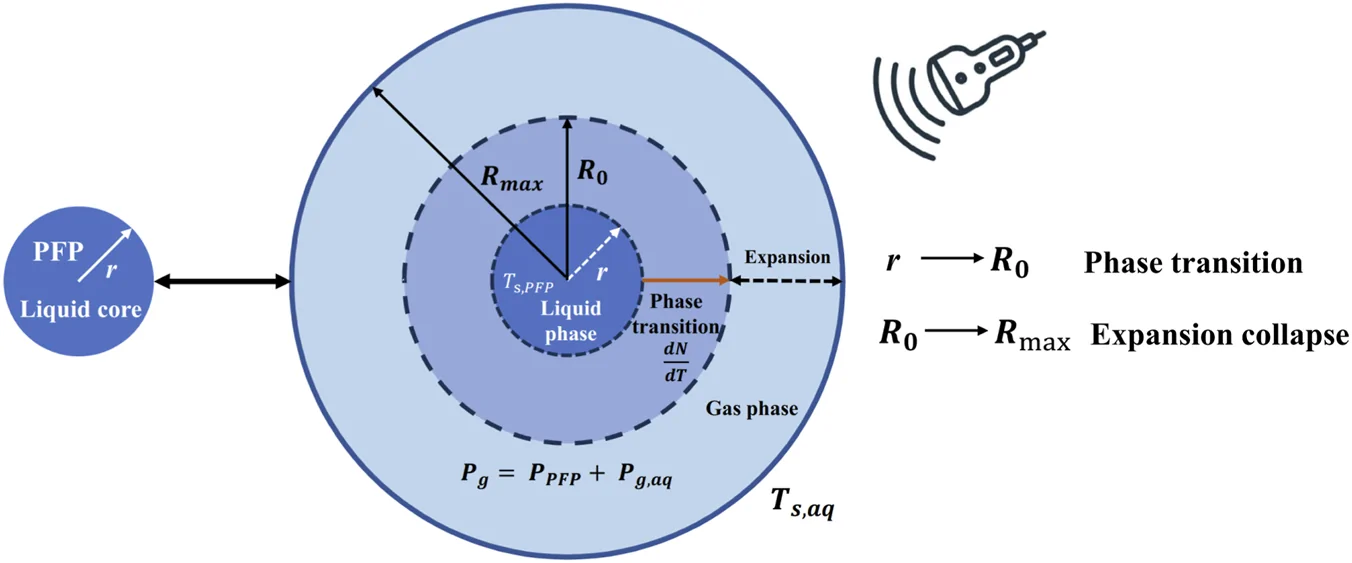
Schematic diagram illustrating the sequential phase transition and cavitation dynamics of PCNDs under ultrasound irradiation. The PFP liquid core undergoes phase transition to form microbubbles, which subsequently expand to $R_{\max}$ ​ and undergo violent cavitation collapse. Key thermodynamic and dynamic parameters during the core process are highlighted.

**FIGURE 3:**
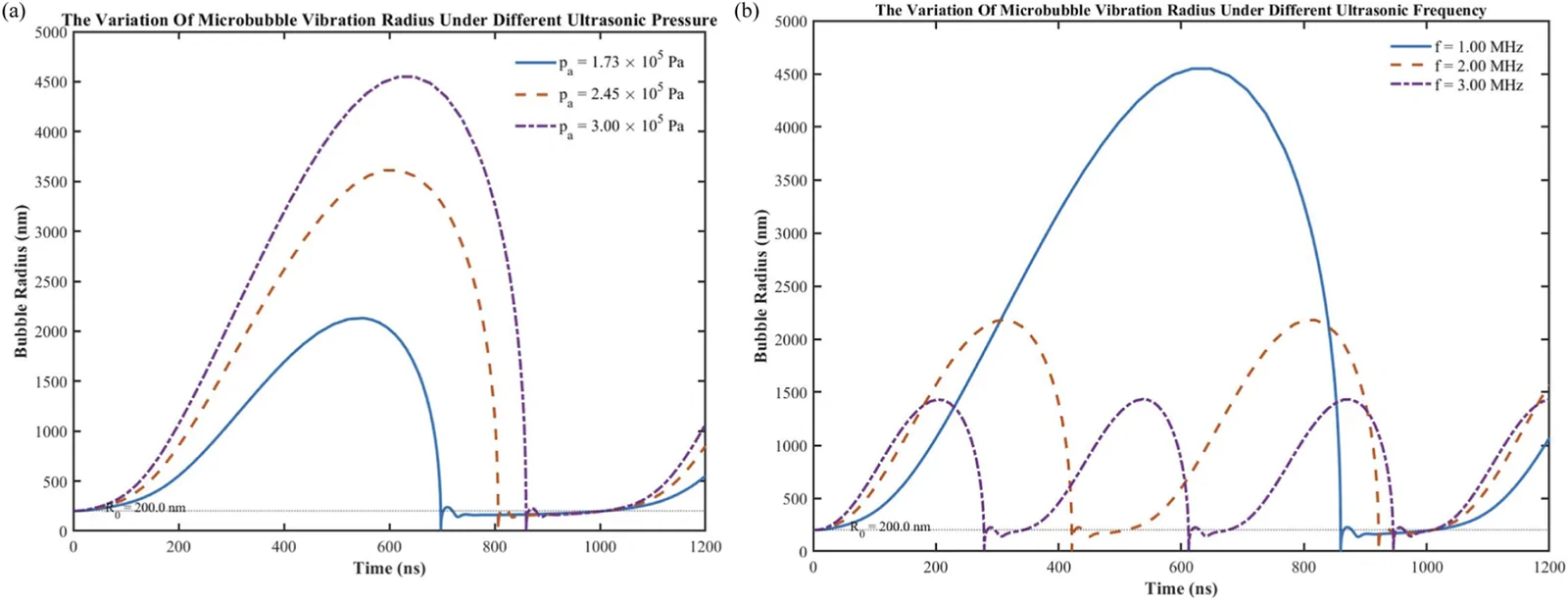
Effects of ultrasonic parameters on the dynamic cavitation behavior of phase-change nanodroplets (PCNDs) simulated by the YCP model. **(a)** Microbubble radius-versus-time curves at a fixed ultrasonic frequency with varying acoustic sound pressures, demonstrating that elevated sound pressure significantly enhances bubble expansion amplitude and cavitation intensity. **(b)** Microbubble radius-versus-time curves at a fixed acoustic sound pressure with varying ultrasonic frequencies, showing that increased frequency shortens the bubble vibration cycle and quenching time but has negligible influence on total cavitation energy output.

Under a fixed frequency of 1 MHz (a clinically relevant parameter for endodontic treatment), the YCP model predicted a significant positive correlation between sound pressure and cavitation efficiency (Figure 4), with specific quantitative results systematically summarized in Table 1. The sound pressure levels tested (2 × 10^5^ Pa, 3 × 10^5^ Pa, 4 × 10^5^ Pa, 6 × 10^5^ Pa, and 9 × 10^5^ Pa) were carefully designed to cover both safe and potentially excessive energy ranges. When the sound pressure increased from 2 × 10^5^ Pa to 3 × 10^5^ Pa and further to 4 × 10^5^ Pa, the cavitation energy showed a steady upward trend, increasing from 7.61 × 10^−14^ J to 8.50 × 10^−14^ J and 1.04 × 10^−13^ J, respectively. Correspondingly, the global expansion ratio rose from 1.1777 to 1.3103 and 1.6017, indicating that higher sound pressure provides a stronger driving force for gas diffusion into the bubble, promoting bubble expansion and enhancing the mechanical energy released during collapse. When the sound pressure exceeded 6 × 10^5^ Pa, a dramatic surge in cavitation energy was observed, with values jumping to 4.23 × 10^−13^ J at 6 × 10^5^ Pa and 4.82 × 10^−13^ J at 9 × 10^5^ Pa. Concurrently, the global expansion ratio exceeded 400, indicating an extremely strong cavitation regime in which inertial cavitation would be expected to occur well before the simulated maximum expansion was reached. Thus, controlling sound pressure within a reasonable clinical range is crucial for balancing therapeutic efficacy and biological safety.

**FIGURE 4:**
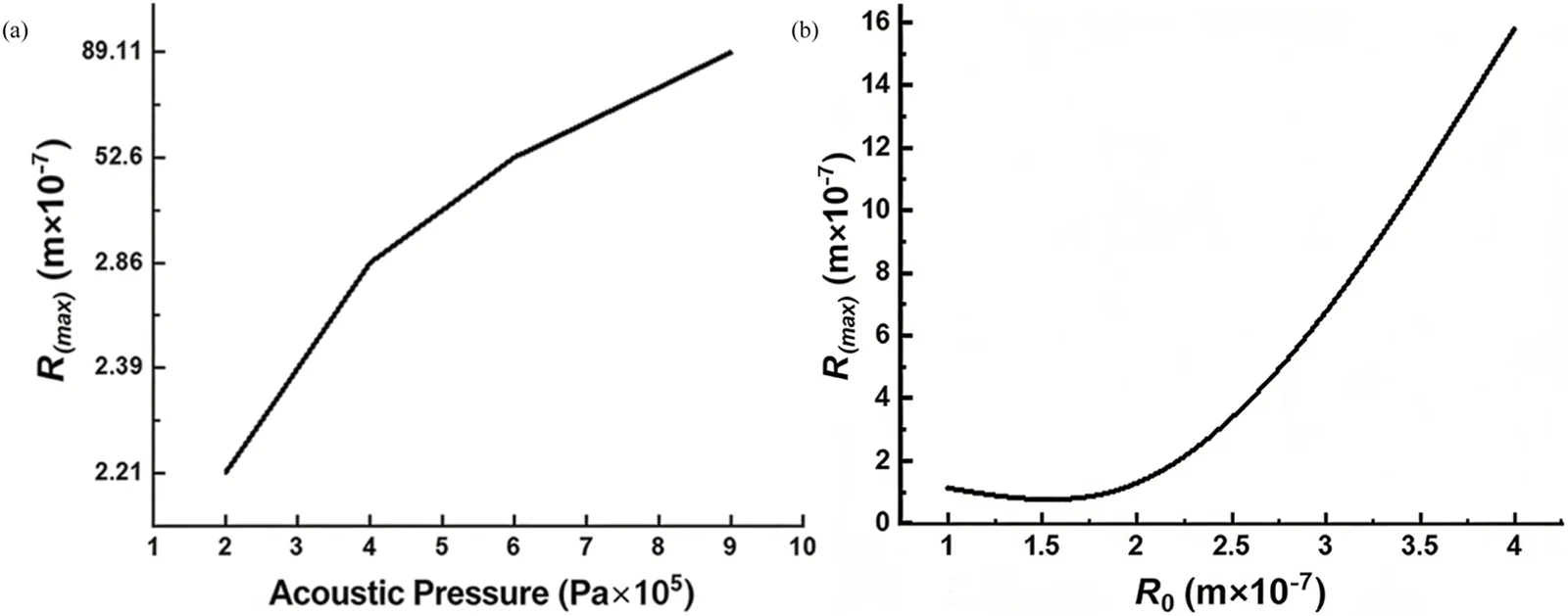
Numerical simulation results of maximum radius of nanodroplet after phase transition cavitation expansion and its correlation with acoustic pressure and initial radius. **(a)** The maximum expansion radius increases exponentially with increasing acoustic pressure. Note that the ordinate is represented exponentially to include the data. **(b)** The correlation between the initial microbubble radius R_0​_ and the maximum expansion radius R_(max)​_ under fixed ultrasonic conditions, revealing a non-monotonic trend: R_(max)​_ remains nearly constant for small initial radii, then grows exponentially with R_0​_ once the threshold is exceeded, highlighting the critical role of initial nanodroplet size in determining cavitation intensity.

**TABLE 1:** Simulation results under different sound pressure conditions (1 MHz frequency).

Sound pressure *p*_1_ (Pa×10^5^)	Cavitation energy $W_{therm}$ (J)	Quench time *t* (s)	*rationglo*	*rationlocal*
2	7.61 × 10^−14^	4.98 × 10^−7^	1.1777	1.1777
3	8.50 × 10^−14^	4.96 × 10^−7^	1.3103	1.3103
4	1.04 × 10^−13^	4.84 × 10^−7^	1.6017	1.6015
6	4.23 × 10^−13^	1.84 × 10^−7^	406.63	406.51
9	4.82 × 10^−13^	2.74 × 10^−7^	867.04	866.36

#### 3.1.2 Effects of material properties on cavitation behavior

The YCP model systematically investigated the influence of key material properties of PCNDs and surrounding media on cavitation behavior, with a focus on parameters that directly affect phase transition and bubble dynamics.

Surface tension, as a core material property regulating the phase transition and cavitation process of PCNDs, was tested at three distinct levels (0.04 N/m, 0.068 N/m, and 0.1 N/m) to simulate different shell compositions and modification strategies, with detailed simulation results summarized in Table 2. When the surface tension was set to 0.04 N/m, the simulated cavitation energy (W) reached 9.99 × 10^−14^ J, the quenching time (t) was 7.29 × 10^−7^ s, and both the global expansion ratio and local expansion ratio were 2.59 (2.5905 and 2.5711, respectively), indicating the most intense cavitation effects due to reduced surface constraints on bubble growth. When the surface tension increased to 0.068 N/m (a value mimicking PCNDs with modified shell components), the cavitation energy decreased to 8.50 × 10^−14^ J, the quenching time shortened to 4.97 × 10^−7^ s, and the expansion ratios reduced to 1.3103, reflecting a weakened cavitation intensity caused by increased surface resistance. A further increase in surface tension to 0.1 N/m resulted in a cavitation energy of 9.36 × 10^−14^ J, a quenching time of 4.98 × 10^−7^ s, and expansion ratios of 1.2525, confirming that excessive surface tension inhibits bubble expansion and limits the mechanical energy released during cavitation.

**TABLE 2:** Simulation results under different surface tension conditions.

Surface tension *σ* (N/m)	Cavitation energy $W_{therm}$ (J)	Quench time *t* (s)	*rationglo*	*rationlocal*
0.04	9.99 × 10^−14^	7.29 × 10^−7^	2.5905	2.5711
0.068	8.50 × 10^−14^	4.97 × 10^−7^	1.3103	1.3103
0.1	9.36 × 10^−14^	4.98 × 10^−7^	1.2525	1.2525

The shear modulus of the surrounding medium, which reflects the viscoelastic properties of dental tissues and biofilms, also played a crucial role in cavitation dynamics. When the shear modulus (G) was increased from 0 MPa (simulating pure water) to 1 MPa and 2 MPa (approaching the shear modulus of dental hard tissue and mature biofilms), the cavitation energy showed a gradual decreasing trend, falling from 8.50 × 10^−14^ J to 7.08 × 10^−14^ J and 6.86 × 10^−14^ J, respectively. Concurrently, the global expansion ratio reduced from 1.3103 to 1.0963 and 1.0585, indicating that the elastic component of the surrounding medium absorbs part of the mechanical energy released during microbubble collapse, thereby weakening cavitation intensity. However, even at a shear modulus of 2 MPa, the cavitation energy remained sufficient to disrupt biofilm structures, confirming the feasibility of the strategy in clinical scenarios.

The initial particle size of PCNDs was another critical material property affecting cavitation behavior. When the initial radius (R_0_) increased from 1 × 10^−7^ m to 2 × 10^−7^ m and further to 4 × 10^−7^ m, the cavitation energy increased significantly by nearly two orders of magnitude, rising from ×1.7210^−14^ J to 8.50 × 10^−14^ J and 9.44 × 10^−13^ J. Correspondingly, the global expansion ratio increased from 1.1397 to 1.3103 and 15.790, attributed to the larger initial volume of PCNDs providing more liquid core material for phase transition, leading to the formation of larger microbubbles and greater energy release during cavitation (Gateau et al., 2011).

In contrast, material properties such as viscosity (0–0.01 Pa·s), sound speed (340–1500 m/s), and density (500–2000 kg/m^3^) showed no significant impact on cavitation energy and expansion ratios, with fluctuations of less than 1% across different levels. Detailed data for these parameters is provided in Supplementary Material, confirming their minimal influence on core cavitation performance.

### 3.2 Experimental results of biofilm elimination

#### 3.2.1 Effects of ultrasonic frequency and intensity on biofilm destruction

Microscope images (Figure 5) showed clear differences in biofilm coverage and morphology between groups. In the blank control group (Group A), the tooth surface was fully covered by a continuous biofilm with intact bacterial chains and a dense extracellular matrix. In contrast, biofilm coverage decreased significantly with increasing ultrasonic intensity for both 1 MHz and 3 MHz frequencies. At an intensity of 1 W/cm^2^ (Group B), partial biofilm detachment was observed, but residual bacterial clusters remained attached to the tooth surface and within dentinal tubules. At 2 W/cm^2^ (Group C), most of the biofilm was removed, with only scattered bacteria left on the tooth surface. At the highest intensity of 3 W/cm^2^ (Group D), the tooth surface was nearly free of biofilm, and the remaining bacteria showed obvious morphological damage, including broken cell walls and fragmented bacterial structures (Flemming et al., 2016).

**FIGURE 5:**
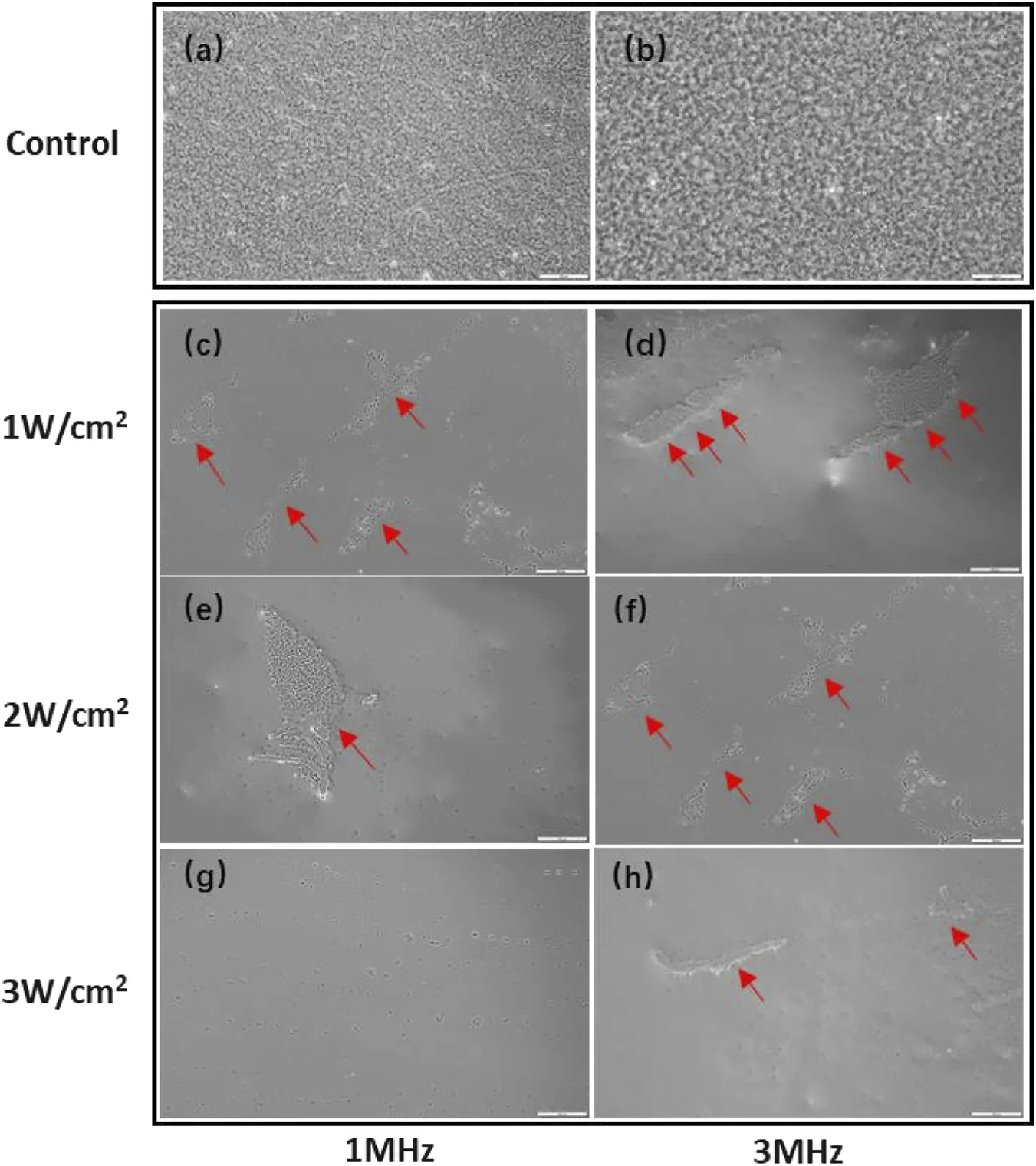
Results of biofilm destruction at different ultrasound frequencies and intensities. **(a,b)** Control group, **(a)** 100-fold magnification, **(b)** 200-fold magnification. **(c)** 1W/cm^2^, 1 MHz. **(d)** 1W/cm^2^, 3 MHz. **(e)** 2W/cm^2^, 1 MHz. **(f)** 2W/cm^2^, 3 MHz. **(g)** 3W/cm^2^, 1 MHz. **(h)** 3W/cm^2^, 3 MHz.

Quantitative analysis of the biofilm removal rate (Figure 6) further confirmed these trends, detailed protocol is provided in Supplementary S3.3. For 1 MHz, the removal rate increased from 66.8% (Group B) to 95.3% (Group D) with increasing intensity. For 3 MHz, the removal rate increased from 69.1% (Group B) to 94.2% (Group D). Statistical analysis showed no significant difference in the removal rate between 1 MHz and 3 MHz at the same intensity (P > 0.05), which is consistent with the simulation results indicating that frequency has no significant impact on total cavitation energy. This finding suggests that ultrasonic intensity is the key parameter regulating biofilm elimination efficacy, while frequency can be adjusted within the clinically relevant range of 1–3 MHz based on equipment availability without compromising treatment outcomes.

**FIGURE 6:**
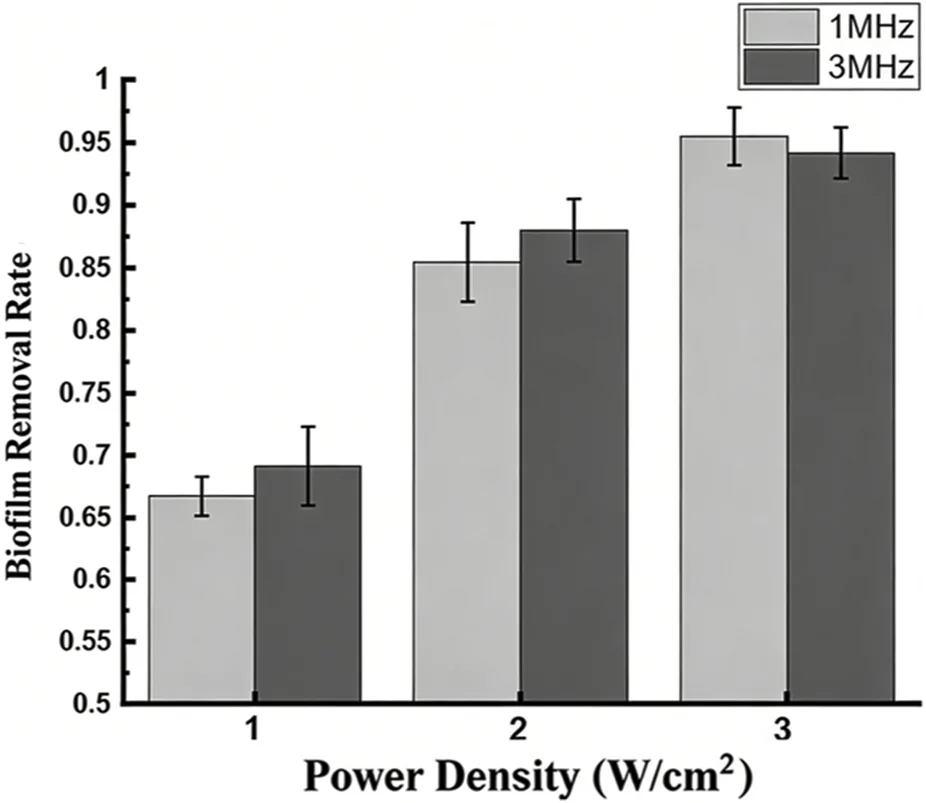
Biofilm removal efficiency under different ultrasonic power densities and frequencies. Biofilm removal rate (%) at various power densities (1, 2, and 3 W/cm^2^) when exposed to 1 MHz (light gray) and 3 MHz (dark gray) ultrasonic waves. The results demonstrate that biofilm eradication increases significantly with higher power density across both frequencies, and 3 MHz ultrasound achieves a similar removal rate with 1 MHz at the same power input. Error bars represent the standard deviation of experimental replicates, highlighting the reliability of the observed enhancement in biofilm elimination efficacy with increased acoustic power.

#### 3.2.2 System temperature changes

Temperature dynamics during ultrasonic treatment were monitored to evaluate the thermal safety of the ultrasound-activated nanodroplet (US + ND) system initiated at 37 °C, with results presented in Figure 7. All systems exhibited a natural temperature decline over 5 min due to ambient heat dissipation, but the US + ND system showed a significantly slower cooling rate, directly reflecting heat released during ultrasonic cavitation and PFP phase transition.

**FIGURE 7:**
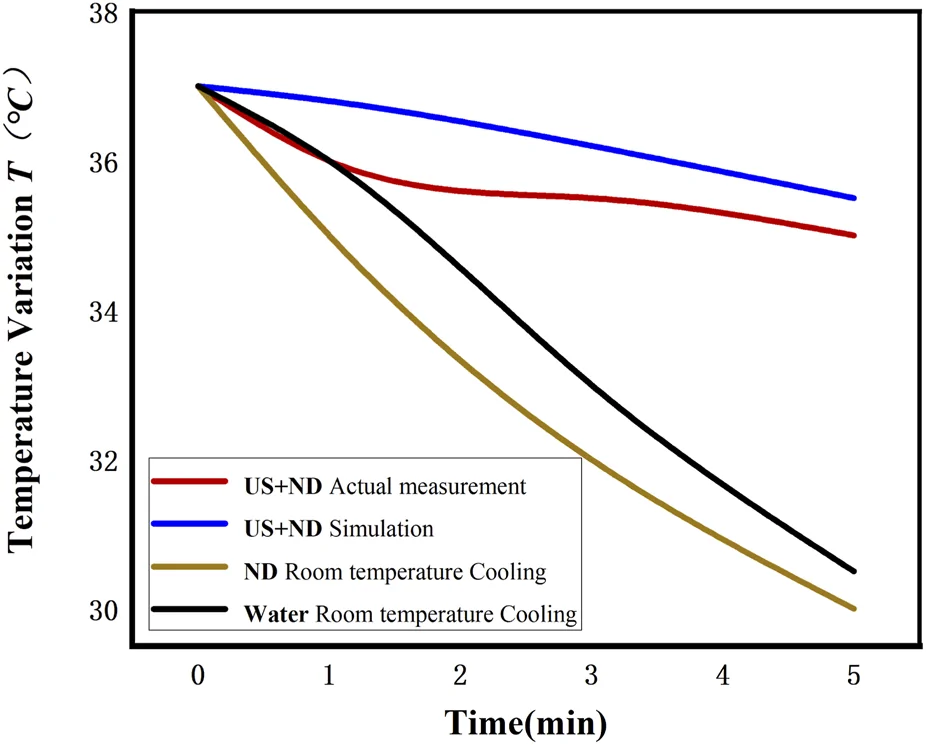
Thermal evolution and cooling performance comparisons. The figure displays the temperature variation (T, °C) over time (min) for four experimental groups: ultrasound combined with nanodroplets (US + ND) measured by actual experiment (red line), US + ND numerical simulation (blue line), nanodroplets-only room-temperature cooling (yellow line), and pure water room-temperature cooling (black line). The results indicate that both water and nanodroplets undergo rapid temperature declines under ambient cooling conditions. The close agreement between the experimental and simulated US + ND curves validates the accuracy of the numerical model in predicting the thermal behavior of ultrasound-activated microbubble systems.

As shown in Figure 7, the room-temperature cooling controls (nanodroplets-only and pure water) experienced rapid, continuous temperature drops, reaching ∼30 °C and ∼30.5 °C at 5 min, respectively. In contrast, the US + ND groups maintained substantially higher temperatures throughout the treatment period. Critically, the simulated US + ND temperature profile (blue line) exhibited excellent agreement with the actual measured data (red line), accurately reproducing the slower cooling trajectory and the magnitude of cavitation-induced thermal buffering observed experimentally. The net temperature elevation relative to the controls was ∼2.0 °C, well below the 5 °C threshold for irreversible dental pulp damage, confirming the excellent thermal safety of this strategy. This close alignment between simulation and experiment validates the reliability of the YCP model in capturing the thermal dynamics of the US + ND system.

### 3.3 Validation of the YCP model (simulation vs. experiment)

The validity and reliability of the YCP model were comprehensively verified by comparing simulated results with experimental data, with key comparisons presented in Figure 8. This validation focused on two core metrics: the relationship between ultrasonic sound pressure, cavitation energy, and biofilm removal rate, and the temporal thermal dynamics of the US + ND system.

**FIGURE 8:**
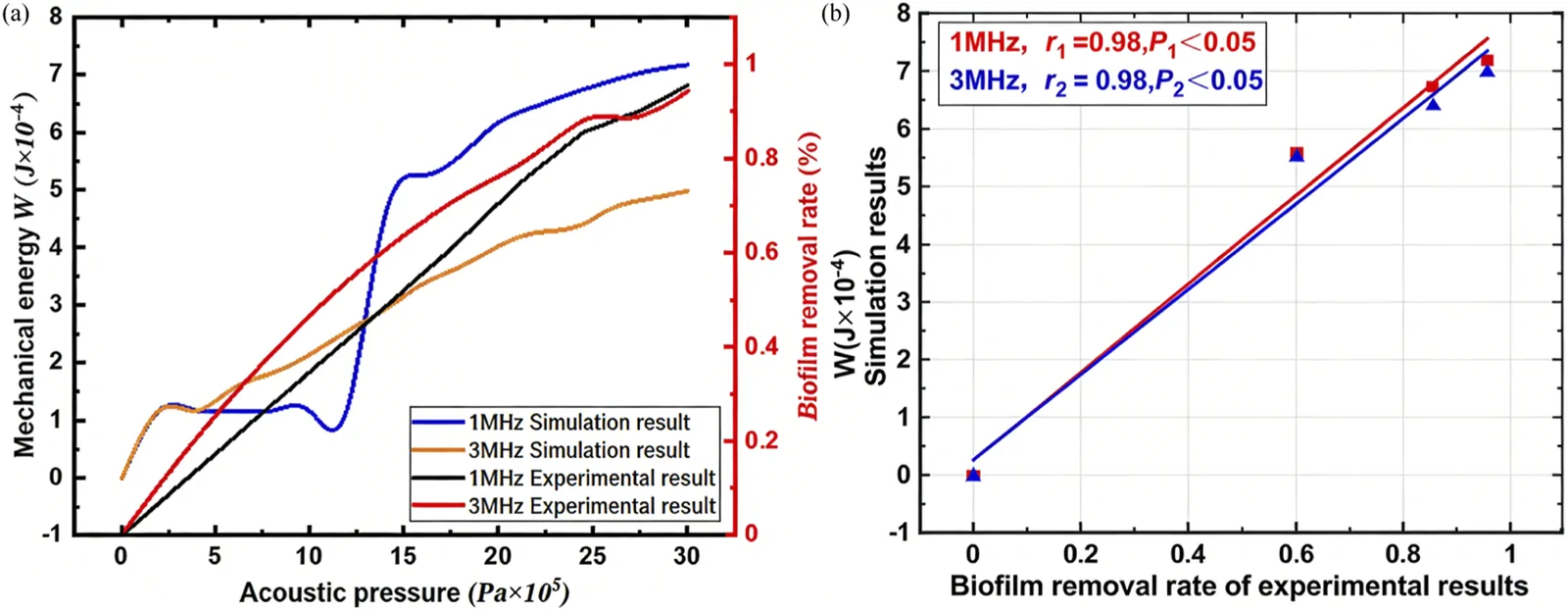
Correlation between mechanical energy and biofilm removal rate. **(a)** Mechanical energy W as function of acoustic pressure. Simulated and experimental data confirm that higher acoustic pressure yields greater energy release and more efficient biofilm elimination, and the simulated and experimental curves exhibit highly consistent trends. **(b)** Linear regression analysis reveals a strong positive correlation (r = 0.988, P = 0.0119 for 1 MHz; r = 0.986, P = 0.0141 for 3 MHz) between simulated energy and experimental removal rate, verifying the accuracy of the model in quantifying cavitation-induced biofilm eradication.

First, the mechanical energy and biofilm removal efficacy were validated (Figure 8A). For both 1 MHz and 3 MHz frequencies, simulated mechanical energy ( $W_{therm}$ ) increased linearly with rising ultrasonic sound pressure. Concurrently, experimental biofilm removal rates showed a parallel upward trend across the same pressure range, with 1 MHz and 3 MHz exhibiting nearly identical removal efficiencies at matched sound pressures. Quantitative correlation analysis (Figure 8B) revealed a strong positive linear relationship between simulated cavitation energy and experimental biofilm removal rate, with Pearson correlation coefficients of r_1_​ = 0.98 (P_1_ < 0.05) for 1 MHz and r_2_ = 0.98 (P_2_​<0.05) for 3 MHz. This close alignment between simulated energy profiles and experimental removal rate trends conclusively validates the YCP model’s ability to accurately predict the mechanical efficacy of ultrasound-activated nanodroplets.

Second, the model’s thermal predictive capability was evaluated by comparing the simulated and measured temperature changes (Figure 7). In the YCP model, temperature evolution was calculated from the Pitt-based interfacial energy balance, considering heat conduction from the surrounding medium and latent heat consumption during PFP vaporization. This thermal equation was solved simultaneously with the phase-transition and bubble-dynamics equations. The model predicted a maximum temperature difference of approximately 1.5 °C, compared with approximately 2.0 °C in the experiment, corresponding to a discrepancy of approximately 0.5 °C.

The experimental data clearly demonstrated that all systems cooled over time, but the US + ND system’s temperature decline was markedly slowed compared to the room-temperature cooling controls. The simulated US + ND temperature trend closely mirrored the actual measured curve, capturing both the overall cooling trajectory and the magnitude of the cavitation-induced thermal buffering. This agreement—where the simulation reproduced the slower decay rate observed in experiments—validates that the YCP model accurately quantifies the heat released during cavitation and phase transition, and its ability to predict the thermal safety profile of the US + ND system.

## 4 Discussion

The biofilm eradication efficacy of ultrasound-activated phase-change nanodroplets (PCNDs) originates from the sequential vaporization-expansion-collapse process driven by the unique physical properties of perfluoropentane (PFP). With a low boiling point of 29.2 °C, PFP readily undergoes liquid-to-gas phase transition once the ultrasonic pressure exceeds the acoustic droplet vaporization (ADV) threshold under the applied experimental conditions, triggering microbubble formation (Lattwein et al., 2020). The 5–6-fold volume expansion during vaporization generates microbubbles appropriately sized to penetrate dentinal tubules, enabling access to biofilms embedded in the complex and narrow root canal anatomy (Durham et al., 2021). The violent expansion and collapse of these microbubbles produce strong shockwaves and microjets, which mechanically disrupt the extracellular matrix of biofilms, detach bacterial colonies, and damage cell structures. The coupled Yang–Church–Pitt (YCP) model integrates phase transition thermodynamics and viscoelastic cavitation dynamics to simulate the entire process. Simulated cavitation energy shows a strong correlation with experimental biofilm elimination efficiency, with a high correlation coefficient consistent with quantitative validation. This result confirms that the YCP model effectively quantifies the cascade from ultrasonic energy input to phase transition, cavitation, and biofilm destruction (Chowdhury et al., 2020; Lattwein et al., 2022).

Material properties and ultrasonic parameters jointly regulate the activation, cavitation behavior, and final therapeutic performance of PCNDs. Surface tension is a key material property that balances phase transition sensitivity and microbubble stability. Lower surface tension reduces the energy barrier for vaporization but weakens cavitation intensity and microbubble durability, whereas excessively high surface tension inhibits phase transition under clinical ultrasound conditions. Therefore, an optimized surface tension range of 0.068–0.075 N/m is recommended to achieve reliable activation and sufficient energy release. In addition, the viscoelasticity of the surrounding dentin environment, represented by the shear modulus of dentin (40 kPa), contributes to partial energy dissipation during cavitation. Compared with water as a medium, the elastic component of dentin absorbs a portion of the mechanical energy generated by microbubble collapse. Nevertheless, the remaining cavitation energy remains adequate for effective biofilm disruption, supporting the feasibility of PCND-based therapy in the actual root canal environment (Sheeran et al., 2012).

Ultrasonic intensity is identified as the most influential parameter determining biofilm removal efficiency. Both simulation and *ex vivo* experimental results demonstrate a clear positive correlation between intensity and elimination rate. At 1 and 3 MHz, the biofilm removal rate increases from approximately 70% at 1 W/cm^2^ to over 92% at 3 W/cm^2^. Higher intensity enhances acoustic pressure amplitude, promoting more extensive microbubble expansion and more violent collapse, thereby strengthening mechanical damage to biofilms. Importantly, even at the highest intensity of 3 W/cm^2^, the system temperature rises by only 2 °C, far below the 5 °C safety threshold for irreversible dental pulp injury, ensuring excellent biosafety and avoiding thermal damage to the pulp and periapical tissues. In contrast, ultrasonic frequency shows no significant effect on therapeutic efficacy at the same intensity. According to the simulation results (Figure 3), increasing frequency accelerates microbubble vibration and shortens collapse time but reduces the maximum bubble radius. The calculated cavitation energies (Supplementary Table S2) indicate that these opposing effects offset each other, resulting in similar total cavitation energy output and comparable biofilm elimination efficiency. This finding allows flexible adjustment of frequency according to clinical equipment without compromising treatment outcomes (Fan et al., 2021).

Thermal dynamic behavior during treatment is highly consistent between model prediction and experimental measurement. The YCP model estimates a maximum temperature increase of 1.5 °C, while experimental detection shows a rise of approximately 2 °C. The slight difference can be attributed to heat loss to the surrounding environment during *ex vivo* testing. The temperature profile follows a rapid-rise-then-stabilization pattern within the treatment duration. In the initial 2 minutes, ultrasonic energy input and endothermic phase transition of PFP reach a dynamic balance, preventing excessive heating (Lau et al., 2023). This controlled temperature elevation provides a significant safety advantage over conventional ultrasonic irrigation, which may cause local overheating and tissue damage.

Based on the combined simulation and experimental results, a safe and effective therapeutic window is established for ultrasound-activated PCNDs: ultrasonic intensity ≤3 W/cm^2^ and frequency ranging from 1 to 3 MHz. Within this window, biofilm removal efficiency exceeds 90% with minimal temperature increase, providing a quantitative and evidence-based reference for clinical parameter selection. Although this study uses unloaded PCNDs, the established YCP model and mechanistic insights provide a solid foundation for future development of drug-loaded PCND systems. Cavitation-induced biofilm disruption can improve the penetration of antimicrobial agents into dentinal tubules, offering a potential strategy to overcome drug resistance in refractory root canal infections.

The simulation trends predicted by the YCP model are generally consistent with previous experimental and theoretical studies on acoustic droplet vaporization and cavitation dynamics. Earlier investigations have demonstrated that increasing acoustic pressure promotes droplet vaporization, enhances cavitation activity, and consequently increases the mechanical disruption of bacterial biofilms, whereas phase-change nanodroplets remain largely inactive before ultrasound activation. These experimentally established mechanisms agree well with the present simulations, in which increasing acoustic pressure produced greater bubble expansion, higher cavitation energy, and enhanced biofilm removal. Such consistency supports the capability of the YCP model to capture the principal physical processes governing ultrasound-activated PCND behavior (Doinikov, 1999). Several limitations exist in the present study. Experiments were performed using mono-species *E. faecalis* biofilms, whereas clinical infections involve complex polymicrobial communities with different structural and physiological characteristics. The YCP model assumes linear ultrasonic propagation, while nonlinear acoustic effects may occur under high-intensity conditions. In addition, the *ex vivo* environment cannot fully replicate the *in vivo* root canal condition with residual pulp, saliva, and periodontal fluids. The current validation primarily confirms the model’s ability to reproduce treatment trends and thermal responses rather than the absolute maximum bubble radius predicted by the simulations. Future studies combining ultra-high-speed optical imaging or calibrated acoustic scattering measurements with the YCP framework will enable more rigorous validation of bubble dynamics and further improve the predictive capability of the model (Reddy and Szeri, 2002). Although acoustic cavitation detection has been well established in previous microbubble and phase-change droplet studies, direct passive or active acoustic measurements were not included in the present study. Therefore, future studies will incorporate acoustic monitoring to further validate the predicted cavitation behavior in the specific PCND–root canal system (Doinikov, 1999).

The present Yang–Church–Pitt (YCP) model should be interpreted as a representative-bubble framework for investigating the local phase-transition process and parameter-dependent mechanical response of vaporized PCNDs rather than as a complete reconstruction of the collective dynamics of the entire activated bubble population (Fuster and Colonius, 2011). The additional simulations showed that bubble–bubble coupling produced measurable quantitative corrections to the absolute oscillation response, particularly at shorter separations, but the principal pressure-dependent trends remained unchanged. Specifically, increasing the incident acoustic pressure consistently enhanced bubble expansion in both the original representative-bubble model and the interaction-corrected model, supporting the mechanistic interpretation presented in this study (Doinikov, 1999; Fuster and Colonius, 2011). Nevertheless, the present model does not account for collective phenomena such as clustering, acoustic shielding, multiple scattering, or complex root canal boundaries.

Future studies will incorporate multi-species biofilms, coupled acoustic-field simulations, and *in vivo* models to further improve the accuracy and translational potential of the proposed strategy.

## 5 Conclusion

In conclusion, this study combined YCP-model simulations with experimental validation to investigate the cavitation behavior of ultrasound-activated phase-change nanodroplets for root canal biofilm eradication. Both simulation and experimental results demonstrated that ultrasound frequency, acoustic intensity, and PCND mechanical properties jointly influence cavitation dynamics, biofilm removal efficiency, and thermal response. The close agreement between the predicted treatment trends and experimental observations supports the applicability of the YCP model for describing the principal physical processes governing ultrasound-activated PCNDs within the investigated parameter range. Although further validation using direct measurements of transient bubble dynamics is warranted, the present work provides a theoretical framework for optimizing ultrasound treatment parameters and offers useful guidance for the rational design and further development of ultrasound-assisted root canal disinfection strategies.

This study proposes a novel strategy for root canal biofilm elimination using ultrasound-activated unloaded phase-change nanodroplets (PCNDs) and establishes a coupled YCP (Yang-Church-Pitt) dynamic model to elucidate its mechanism. This work demonstrates that ultrasound-activated unloaded PCNDs are a safe and effective approach for root canal biofilm elimination, while the YCP model provides a theoretical tool for optimizing treatment parameters. Future research will address mixed-species biofilms and *in vivo* validation, further advancing the clinical translation of this technology.

## Data Availability

The original contributions presented in the study are included in the article/Supplementary Material, further inquiries can be directed to the corresponding author.
