# Supplementary material for: Ultrasound-activated phase-change nanodroplets for root canal biofilm eradication: mechanistic Investigation and experimental validation
Source: Front Bioeng Biotechnol. 2026 Oct 28;14:1882780. doi: 10.3389/fbioe.2026.1882780 (PMC13751481; doi:10.3389/fbioe.2026.1882780)
Supplement: Supplementary file 1 [file Supplementaryfile1.docx]

Supplementary Material

# 1 Nomenclature, Parameter Values, and Derivation Details

## 1.1 Parameter Definitions and Default Values

All parameters utilized in the Yang-Church-Pitt (YCP) dynamic model, including their physical meanings, symbols, and numerical values adopted for simulations, are summarized in Table S1. These parameters were selected based on experimental conditions, material properties, and literature references to ensure the model’s accuracy and relevance to root canal biofilm treatment scenarios.

**Table S1.** List of parameters, symbols, and values in the YCP model.

| Category | Parameter Name | Symbol | Value | Unit |
| --- | --- | --- | --- | --- |
| Ultrasonic Parameters | Ultrasonic frequency | f_1_ | 0.5×10^6^–2×10^6^ | Hz |
|  | Ultrasonic pressure | p_1_ | 1.73×10⁵–9×10⁵ | Pa |
|  | Speed of sound in medium | c | 340–1500 | m/s |
| Material Properties (PCNDs) | Initial nanodroplet radius | R_0_ | 1×10⁻⁷–4×10⁻⁷ | m |
|  | Surface tension | σ | 0.04–0.1 | N/m |
|  | Molecular weight of PFP | M_PFP_ | 462 |  |
|  | Specific heat capacity of PFP | C_m,PFP_ | 1.38 | J/(kg·K) |
|  | Average thermal conductivity of PFP | k_gas_ | 0.018 | W/(m·K) |
|  | Enthalpy of vaporization of PFP | ΔH_PFP_ | 43.3×10^3^ | J/mol |
| Material Properties (Medium) | Density of medium | ρ | 500–2000 | kg/m^3^ |
|  | Viscosity of medium | μ | 0–0.01 | Pa*s |
|  | Shear modulus of medium | G | 0–2 | MPa |
| Thermodynamic Parameters | Ideal gas constant | Γ | 8.314 | J/(mol·K) |
|  | Atmospheric pressure | p_0_ | 1.013×10⁵ | Pa |
|  | Initial temperature of medium | T_s,aq_ | 293 | K |
|  | Polytropic exponent | κ | 1.4 | - |

## 1.2 Derivation of the Microbubble Interface Dynamic Model

Taking the perspective of a single microbubble, the continuity equation in fluid mechanics is first applied in a spherical coordinate system, yielding:

$$\begin{aligned} \frac{\boldsymbol{\partial\rho}}{\boldsymbol{\partial t}}\boldsymbol{+}\frac{\boldsymbol{\partial}\left( \boldsymbol{\rho}\boldsymbol{v}_{\boldsymbol{r}} \right)}{\boldsymbol{\partial r}}\boldsymbol{+}\frac{\boldsymbol{2}\boldsymbol{\rho}\boldsymbol{v}_{\boldsymbol{r}}}{\boldsymbol{r}}\boldsymbol{=0\#}\left( \boldsymbol{S}\boldsymbol{1} \right) \end{aligned}$$

Then, considering the conservation of radial momentum in spherical coordinates:

$$\begin{aligned} \boldsymbol{\rho}\left( \frac{\boldsymbol{\partial}\boldsymbol{v}_{\boldsymbol{r}}}{\boldsymbol{\partial t}}\boldsymbol{+}\boldsymbol{v}_{\boldsymbol{r}}\frac{\boldsymbol{\partial}\boldsymbol{v}_{\boldsymbol{r}}}{\boldsymbol{\partial t}} \right)\boldsymbol{=-}\frac{\boldsymbol{\partial p}}{\boldsymbol{\partial r}}\boldsymbol{+}\frac{\boldsymbol{\partial}\boldsymbol{\tau}_{\boldsymbol{r}}}{\boldsymbol{\partial r}}\boldsymbol{+}\frac{\boldsymbol{2}}{\boldsymbol{r}}\left( \boldsymbol{\tau}_{\boldsymbol{r}}\boldsymbol{-}\boldsymbol{\tau}_{\boldsymbol{\theta}} \right)\boldsymbol{\#}\left( \boldsymbol{S}\boldsymbol{2} \right) \end{aligned}$$

Next, the boundary and initial conditions are given as:

$$\begin{aligned} \boldsymbol{p=}\boldsymbol{p}_{\boldsymbol{g}}\boldsymbol{-}\frac{\boldsymbol{2}\boldsymbol{\sigma}}{\boldsymbol{R}}\boldsymbol{+}\boldsymbol{\tau}_{\boldsymbol{r}}\boldsymbol{，}\boldsymbol{r=R\#}\left( \boldsymbol{S}\boldsymbol{3} \right) \end{aligned}$$

The above equations are insufficient for solution, and approximations for the near-field and far-field are required. According to the prior assumptions of Yang and Church, the near-field motion of the microbubble is dominated by its own compression and expansion, and the surrounding medium is nearly incompressible. Thus, the internal radial pressure of the bubble can be obtained by synthesizing the Bernoulli momentum equation:

$$\begin{aligned} \boldsymbol{p}_{\boldsymbol{in}}\boldsymbol{=}\boldsymbol{p}_{\boldsymbol{a}}\boldsymbol{-}\boldsymbol{\rho}_{\boldsymbol{0}}\left( \boldsymbol{R}\boldsymbol{a}_{\boldsymbol{R}}\boldsymbol{+}\frac{\boldsymbol{3}}{\boldsymbol{2}}\boldsymbol{v}_{\boldsymbol{R}}^{\boldsymbol{2}} \right)\boldsymbol{+}\frac{\boldsymbol{\rho}_{\boldsymbol{0}}}{\boldsymbol{r}}\frac{\text{d}\left( \boldsymbol{R}^{\boldsymbol{2}}\boldsymbol{v}_{\boldsymbol{R}} \right)}{\text{d}\boldsymbol{t}}\boldsymbol{-}\frac{\boldsymbol{\rho}_{\boldsymbol{0}}}{\boldsymbol{2}}\frac{\boldsymbol{R}^{\boldsymbol{4}}\boldsymbol{a}_{\boldsymbol{R}}^{\boldsymbol{2}}}{\boldsymbol{r}^{\boldsymbol{4}}}\boldsymbol{+}\boldsymbol{\tau}_{\boldsymbol{r}}\boldsymbol{|}_{\boldsymbol{R}}^{\boldsymbol{r}}\boldsymbol{+3}\int_{\boldsymbol{R}}^{\boldsymbol{r}} \frac{\boldsymbol{\tau}_{\boldsymbol{r}}}{\boldsymbol{r}}\text{d}\boldsymbol{r}\boldsymbol{\#}\left( \boldsymbol{S}\boldsymbol{4} \right) \end{aligned}$$

In the far-field (sufficiently far from the microbubble), the pressure and density fluctuations of the liquid are small, and the stress and convection terms are negligible. Only the force exerted by the external sound field is considered, and the linear acoustic equation is adopted:

$$\begin{aligned} \boldsymbol{\phi}_{\boldsymbol{ex}}\boldsymbol{=}\frac{\boldsymbol{1}}{\boldsymbol{r}}\left[ \boldsymbol{\psi}_{\boldsymbol{1}}\left( \boldsymbol{t-}\frac{\boldsymbol{r}}{\boldsymbol{c}} \right)\boldsymbol{+}\boldsymbol{\psi}_{\boldsymbol{2}}\left( \boldsymbol{t+}\frac{\boldsymbol{r}}{\boldsymbol{c}} \right) \right]\boldsymbol{\#}\left( \boldsymbol{S}\mathbf{5} \right) \end{aligned}$$

$$\begin{aligned} \boldsymbol{p}_{\boldsymbol{ex}}\boldsymbol{=}\boldsymbol{p}_{\boldsymbol{0}}\boldsymbol{-}\boldsymbol{\rho}_{\boldsymbol{0}}\frac{\boldsymbol{\partial}\boldsymbol{\phi}_{\boldsymbol{ex}}}{\boldsymbol{\partial t}}\boldsymbol{\#}\left( \boldsymbol{s}\boldsymbol{6} \right) \end{aligned}$$

With the approximate prior knowledge of both near-field and far-field, to obtain the radial motion equation of the bubble under a given driving pressure, the approximate equations of near-field and far-field can be unified to solve the asymptotically matched solution. The matching conditions are equal volume flow rate and pressure:

$$\begin{aligned} {\boldsymbol{4}\boldsymbol{\pi}\boldsymbol{r}^{\boldsymbol{2}}\boldsymbol{v}_{\boldsymbol{rin}}\left. \boldsymbol{} \right|}_{\boldsymbol{r\to\infty}}\boldsymbol{=}{\boldsymbol{4}\boldsymbol{\pi}\boldsymbol{r}^{\boldsymbol{2}}\boldsymbol{v}_{\boldsymbol{rex}}\left. \boldsymbol{} \right|}_{\boldsymbol{r\to0}}\boldsymbol{\#}\left( \boldsymbol{S}\boldsymbol{7} \right) \end{aligned}$$

$$\begin{aligned} {\boldsymbol{p}_{\boldsymbol{in}}\left. \boldsymbol{} \right|}_{\boldsymbol{r\to\infty}}\boldsymbol{=}{\boldsymbol{p}_{\boldsymbol{ex}}\left. \boldsymbol{} \right|}_{\boldsymbol{r\to0}}\boldsymbol{\#}\left( \boldsymbol{S}\boldsymbol{8} \right) \end{aligned}$$

After solving formulas simultaneously, there is:

$$\begin{aligned} \boldsymbol{R}\boldsymbol{a}_{\boldsymbol{R}}\boldsymbol{+}\frac{\boldsymbol{3}}{\boldsymbol{2}}\boldsymbol{R}^{\boldsymbol{2}}\boldsymbol{=}\frac{\boldsymbol{p}_{\boldsymbol{a}}\boldsymbol{-}\boldsymbol{p}_{\boldsymbol{0}}}{\boldsymbol{\rho}}\boldsymbol{+}\frac{\boldsymbol{1}}{\boldsymbol{c}}\left( \boldsymbol{2}\boldsymbol{\psi}_{\boldsymbol{2}}^{\boldsymbol{‘’}}\boldsymbol{+}\frac{\boldsymbol{d}^{\text{2}}\boldsymbol{Q}}{\text{d}\boldsymbol{t}^{\boldsymbol{2}}} \right)\boldsymbol{-}\frac{\boldsymbol{\tau}_{\boldsymbol{r}}\left( \boldsymbol{R} \right)}{\boldsymbol{\rho}}\boldsymbol{+}\frac{\boldsymbol{3}}{\boldsymbol{\rho}}\int_{\boldsymbol{R}}^{\boldsymbol{\infty}} \frac{\boldsymbol{\tau}_{\boldsymbol{r}}}{\boldsymbol{r}}\text{d}\boldsymbol{r\#}\left( \boldsymbol{S}\boldsymbol{9} \right) \end{aligned}$$

Neglect the high-order infinitesimal according to the prior assumptions of Yang and Church, there is:

$$\begin{aligned} \left( \boldsymbol{1-}\frac{\boldsymbol{v}_{\boldsymbol{R}}}{\boldsymbol{c}} \right)\boldsymbol{R}\boldsymbol{a}_{\boldsymbol{R}}\boldsymbol{+}\frac{\boldsymbol{3}}{\boldsymbol{2}}\left( \boldsymbol{1-}\frac{\boldsymbol{v}_{\boldsymbol{R}}}{\boldsymbol{3}\boldsymbol{c}} \right)\boldsymbol{v}_{\boldsymbol{R}}^{\boldsymbol{2}}\boldsymbol{=}\left( \boldsymbol{1+}\frac{\boldsymbol{v}_{\boldsymbol{R}}}{\boldsymbol{c}} \right)\frac{\boldsymbol{p}_{\boldsymbol{a}}\boldsymbol{-}\boldsymbol{p}_{\boldsymbol{I}}}{\boldsymbol{\rho}}\boldsymbol{+}\frac{\boldsymbol{R}\text{d}\left( \boldsymbol{p}_{\boldsymbol{a}}\boldsymbol{-}\boldsymbol{p}_{\boldsymbol{I}} \right)}{\boldsymbol{\rho c}\text{d}\boldsymbol{t}}\boldsymbol{\#}\left( \boldsymbol{S}\boldsymbol{10} \right) \end{aligned}$$

Next, supplement all information of the undefined variables, where p_a_ is the surface pressure of the microbubble:

$$\begin{aligned} \boldsymbol{p}_{\boldsymbol{a}}\boldsymbol{=}\boldsymbol{p}_{\boldsymbol{g}}\boldsymbol{-}\frac{\boldsymbol{2}\boldsymbol{\sigma}}{\boldsymbol{R}}\boldsymbol{+}\boldsymbol{\tau}_{\boldsymbol{r}}\left( \boldsymbol{R} \right)\boldsymbol{\#}\left( \boldsymbol{S}\boldsymbol{11} \right) \end{aligned}$$

$$\begin{aligned} \boldsymbol{p}_{\boldsymbol{I}}\boldsymbol{=}\boldsymbol{p}_{\boldsymbol{0}}\boldsymbol{-}\frac{\boldsymbol{2}\boldsymbol{\rho}\boldsymbol{\psi}_{\boldsymbol{2}}^{\boldsymbol{‘’}}}{\boldsymbol{c}}\boldsymbol{+}\boldsymbol{\tau}_{\boldsymbol{r}}\left( \boldsymbol{R} \right)\boldsymbol{-3}\int_{\boldsymbol{R}}^{\boldsymbol{\infty}} \frac{\boldsymbol{\tau}_{\boldsymbol{r}}}{\boldsymbol{r}}\text{d}\boldsymbol{r\#}\left( \boldsymbol{S}\boldsymbol{12} \right) \end{aligned}$$

Then the integral of shear stress is obtained, along with the equation of p_g_ varying with the microbubble size, where κ is the polytropic index reflecting the relationship between air density and pressure changes inside the bubble:

$$\begin{aligned} \boldsymbol{p}_{\boldsymbol{g}}\boldsymbol{=}\boldsymbol{p}_{\boldsymbol{g}_{\boldsymbol{0}}}\left( \frac{\boldsymbol{R}_{\boldsymbol{0}}}{\boldsymbol{R}} \right)^{\boldsymbol{3}\boldsymbol{\kappa}}\boldsymbol{\#}\left( \boldsymbol{S}\boldsymbol{13} \right) \end{aligned}$$

The excitation signal sound pressure PAg(t) is defined as follows, where p_1_ is the ultrasonic sound pressure (amplitude):

$$\begin{aligned} \frac{\boldsymbol{2}\boldsymbol{\rho}\boldsymbol{\psi}_{\boldsymbol{2}}^{\boldsymbol{‘’}}}{\boldsymbol{c}}\boldsymbol{=}\boldsymbol{P}_{\boldsymbol{A}}\boldsymbol{g}\left( \boldsymbol{t} \right)\boldsymbol{\#}\left( \boldsymbol{S}\boldsymbol{14} \right) \end{aligned}$$

$$\begin{aligned} \boldsymbol{P}_{\boldsymbol{A}}\boldsymbol{g}\left( \boldsymbol{t} \right)\boldsymbol{=}\boldsymbol{p}_{\boldsymbol{1}}\boldsymbol{sin}\left( \boldsymbol{2}\boldsymbol{\pi}\boldsymbol{f}_{\boldsymbol{1}}\boldsymbol{t} \right)\boldsymbol{\#}\left( \boldsymbol{S}\mathbf{15} \right) \end{aligned}$$

After obtaining the above results, as mentioned in Yang’s paper, an analytical solution can be obtained by making a further approximation assuming a small vibration amplitude:

$$\begin{aligned} \boldsymbol{R}^{\boldsymbol{-3}\boldsymbol{\kappa}}\mathbf{=}\boldsymbol{R}_{\boldsymbol{0}}^{\boldsymbol{-3}\boldsymbol{\kappa}}\left( \boldsymbol{1-3}\boldsymbol{\kappa} \right)\boldsymbol{\#}\left( \boldsymbol{S}\boldsymbol{16} \right) \end{aligned}$$

And we now get:

$$\begin{aligned} \frac{\text{d}\left( \boldsymbol{p}_{\boldsymbol{a}}\boldsymbol{-}\boldsymbol{p}_{\boldsymbol{I}} \right)}{\boldsymbol{dt}}\boldsymbol{=}\left[ \frac{\boldsymbol{2}\boldsymbol{\sigma}}{\boldsymbol{R}}\boldsymbol{-3}\boldsymbol{\kappa}\boldsymbol{p}_{\boldsymbol{g}_{\boldsymbol{0}}}\left( \frac{\boldsymbol{R}_{\boldsymbol{0}}}{\boldsymbol{R}} \right)^{\boldsymbol{3}\boldsymbol{\kappa}} \right]\frac{\boldsymbol{v}_{\boldsymbol{R}}}{\boldsymbol{R}}\boldsymbol{+}\boldsymbol{P}_{\boldsymbol{A}}\frac{\text{d}\boldsymbol{g}\left( \boldsymbol{t} \right)}{\text{d}\boldsymbol{t}}\boldsymbol{-4}\boldsymbol{G}\frac{\boldsymbol{R}_{\boldsymbol{0}}^{\boldsymbol{3}}\boldsymbol{v}_{\boldsymbol{R}}}{\boldsymbol{R}^{\boldsymbol{4}}}\boldsymbol{-4}\boldsymbol{\mu}\left( \frac{\boldsymbol{a}_{\boldsymbol{R}}}{\boldsymbol{R}}\boldsymbol{-}\frac{\boldsymbol{v}_{\boldsymbol{R}}^{\boldsymbol{2}}}{\boldsymbol{R}^{\boldsymbol{2}}} \right)\boldsymbol{\#}\left( \boldsymbol{S}\boldsymbol{17} \right) \end{aligned}$$

The above is the microbubble interface dynamic model. For energy conservation, the work done by the external pressure on the microbubble against the internal pressure is the kinetic energy gained by the liquid.

The total mass of the phase transition is the sum of the mass change caused by microbubble vibration and the part affected by temperature during vibration.

$$\begin{aligned} \frac{\boldsymbol{dN}}{\boldsymbol{dt}}\mathbf{=-}\frac{\boldsymbol{d}\boldsymbol{m}_{\boldsymbol{PFP}}\mathbf{+}\boldsymbol{d}\boldsymbol{m}_{\boldsymbol{aq}}}{\bar{\boldsymbol{M}_{\boldsymbol{w}}}\boldsymbol{dt}}\boldsymbol{\#}\left( \boldsymbol{S}\boldsymbol{18} \right) \end{aligned}$$

The composition ratio of the encapsulating bubble membrane is nearly constant, and the displacement and phase transition relationship caused by temperature-dependent bubble density are considered.

$$\begin{aligned} \boldsymbol{\rho}_{\boldsymbol{g}}\mathbf{4}\boldsymbol{\pi}\boldsymbol{R}^{\mathbf{2}}\frac{\boldsymbol{dR}}{\boldsymbol{dt}}\mathbf{-}\frac{\mathbf{4}}{\mathbf{3}}\boldsymbol{\pi}\left( \boldsymbol{R}^{\mathbf{3}}\mathbf{-}\boldsymbol{R}_{\boldsymbol{L}}^{\mathbf{3}} \right)\left( \frac{\boldsymbol{p}_{\boldsymbol{g}}\bar{\boldsymbol{M}_{\boldsymbol{w}}}}{\boldsymbol{\Gamma}{\bar{\boldsymbol{T}_{\boldsymbol{g}}}}^{\mathbf{2}}} \right)\frac{\boldsymbol{d}\bar{\boldsymbol{T}_{\boldsymbol{g}}}}{\boldsymbol{dt}}\mathbf{=}\left( \bar{\boldsymbol{M}_{\boldsymbol{w}}}\mathbf{-}\frac{\boldsymbol{\rho}_{\boldsymbol{g}}}{\boldsymbol{\rho}_{\boldsymbol{PFP}}}\boldsymbol{X}_{\boldsymbol{PFP}}\boldsymbol{M}_{\boldsymbol{PFP}} \right)\frac{\boldsymbol{dN}}{\boldsymbol{dt}}\boldsymbol{\#}\left( \boldsymbol{S}\boldsymbol{19} \right) \end{aligned}$$

And now we consider energy conservation in the phase transition process. Considering the interface between the PFP droplet and the gas phase, the sum of heat conduction, thermal radiation (negligible), and vaporization phase transition is the heat absorption and release change of the droplet.

The same result as the main text is obtained as follows:

$$\begin{aligned} \frac{\boldsymbol{d}\boldsymbol{T}_{\boldsymbol{droplet}}}{\boldsymbol{dt}}\mathbf{=}\frac{\mathbf{3}}{\boldsymbol{\rho}\boldsymbol{C}_{\boldsymbol{m}\mathbf{,}\boldsymbol{PFP}}\boldsymbol{R}_{\boldsymbol{L}}}\left[ \boldsymbol{\theta}\frac{\boldsymbol{k}_{\boldsymbol{gas}}}{\boldsymbol{R}_{\boldsymbol{L}}}\left( \boldsymbol{T}_{\boldsymbol{s}\mathbf{,}\boldsymbol{aq}}\mathbf{-}\boldsymbol{T}_{\boldsymbol{s}\mathbf{,}\boldsymbol{PFP}} \right)\mathbf{-}\frac{\boldsymbol{X}_{\boldsymbol{PFP}}\boldsymbol{M}_{\boldsymbol{PFP}}{\boldsymbol{\Delta}\boldsymbol{H}}_{\boldsymbol{PFP}}}{\mathbf{4}\boldsymbol{\pi}\boldsymbol{R}_{\boldsymbol{L}}^{\mathbf{2}}}\frac{\boldsymbol{dN}}{\boldsymbol{dt}} \right]\boldsymbol{\#}\left( \boldsymbol{S}\mathbf{20} \right) \end{aligned}$$

We can now solve the simultaneous equations of (S20), (S19), and (S17) as we did in the main text.

# 2 Supplementary Simulation Results of Parameter Variations

Additional simulation results for a full set of material and ultrasonic parameters are presented in Tables S2–S7. Among these, sound speed (*c*) and medium density (*ρ*) show negligible influence on cavitation performance, with almost no change in core metrics including cavitation energy, global/local expansion ratio, and quench time. Medium viscosity (μ) also has only minor effects. In contrast, initial nanodroplet radius (*R_0_*), shear modulus (*G*), surface tension (*σ*), and ultrasonic frequency (*f*) produce clear, systematic variations in cavitation behavior. Together, these extended results verify the robustness of the YCP model and clarify key versus secondary parameters for optimizing biofilm disruption.

**Table S2.** Simulation results under different ultrasonic frequency conditions.

| Frequency $f$(Hz) | Cavitation energy $W_{therm}$ (J) | Quench time *t* (s) | *rationglo* | *rationlocal* |
| --- | --- | --- | --- | --- |
| 0.5×10^6^ | 8.49×10^-14^ | 9.97×10^-7^ | 1.3097 | 1.3097 |
| 1×10^6^ | 8.50×10^-14^ | 4.96×10^-7^ | 1.3103 | 1.3103 |
| 2×10^6^ | 8.52×10^-14^ | 2.45×10^-7^ | 1.3128 | 1.3128 |

**Table S3.** Simulation results under different ultrasonic speed conditions.

| Speed $c$(m/s) | Cavitation energy $W_{therm}$ (J) | Quench time *t* (s) | *rationglo* | *rationlocal* |
| --- | --- | --- | --- | --- |
| 340 | 8.50×10^-14^ | 4.97×10^-7^ | 1.3103 | 1.3103 |
| 1000 | 8.50×10^-14^ | 4.97×10^-7^ | 1.3103 | 1.3103 |
| 1500 | 8.50×10^-14^ | 4.96×10^-7^ | 1.3103 | 1.3103 |

**Table S4.** Simulation results under different initial radius of PFP conditions.

| Initial Radius $R_{0}$(m) | Cavitation energy $W_{therm}$ (J) | Quench time *t* (s) | *rationglo* | *rationlocal* |
| --- | --- | --- | --- | --- |
| 1×10^-7^ | 1.72×10^-14^ | 4.99×10^-7^ | 1.1397 | 1.1397 |
| 2×10^-7^ | 8.50×10^-14^ | 4.97×10^-7^ | 1.3103 | 1.3103 |
| 4×10^-7^ | 9.44×10^-13^ | 9.60×10^-7^ | 15.790 | 15.782 |

**Table S5.** Simulation results under different medium density conditions.

| Medium Density $\rho$(𝑘𝑔/𝑚^3^) | Cavitation energy $W_{therm}$ (J) | Quench time *t* (s) | *rationglo* | *rationlocal* |
| --- | --- | --- | --- | --- |
| 500 | 8.49×10^-14^ | 4.96×10^-7^ | 1.3098 | 1.3098 |
| 1000 | 8.50×10^-14^ | 4.97×10^-7^ | 1.3103 | 1.3103 |
| 2000 | 8.51×10^-14^ | 4.97×10^-7^ | 1.3113 | 1.3113 |

**Table S6.** Simulation results under different medium viscosity conditions.

| Viscosity 𝜇(Pa·s) | Cavitation energy $W_{therm}$ (J) | Quench time *t* (s) | *rationglo* | *rationlocal* |
| --- | --- | --- | --- | --- |
| 0 | 8.50×10^-14^ | 4.85×10^-7^ | 1.3107 | 1.3092 |
| 0.01 | 8.50×10^-14^ | 4.97×10^-7^ | 1.3103 | 1.3103 |
| 0.001 | 8.42×10^-14^ | 4.75×10^-7^ | 1.3010 | 1.3010 |

**Table S7.** Simulation results under different medium shear modulus conditions.

| Shear Modulus$G$(MPa) | Cavitation energy $W_{therm}$ (J) | Quench time *t* (s) | *rationglo* | *rationlocal* |
| --- | --- | --- | --- | --- |
| 0 | 8.50×10^-14^ | 4.97×10^-7^ | 1.3103 | 1.3103 |
| 1 | 7.08×10^-14^ | 5.00×10^-7^ | 1.0963 | 1.0963 |
| 2 | 6.86×10^-14^ | 5.00×10^-7^ | 1.0585 | 1.0585 |

# 3 Supplementary Experimental Details

## 3.1 Fabrication and Characterization of PCNDs

PCNDs were prepared using a modified thin-film hydration method followed by probe sonication. Perfluoropentane (PFP) was used as the phase-change liquid core, while the composite interfacial stabilizing layer consisted of DSPC, DSPE-PEG-2000, and Pluronic F-68. Briefly, 10 mg of DSPC, 4 mg of DSPE-PEG-2000, and 8.7 mg of Pluronic F-68 were dissolved in 2 mL of ethanol in a 250-mL round-bottom flask. The solvent was removed by rotary evaporation at 55 °C for 20 min to form a uniform thin lipid film on the inner wall of the flask. The film was subsequently hydrated with 2 mL of a PBS/glycerol solution (9:1, v/v) at 70 °C for 20 min to obtain a lipid precursor suspension.

The precursor suspension was transferred into a sealed glass vial maintained in an ice bath. Subsequently, 200 μL of liquid PFP was added, and the vial was immediately sealed to minimize spontaneous evaporation. Probe sonication was then performed for 5 min at 25% output power with a 50% duty cycle, with the probe tip positioned below the liquid surface to generate PFP-core nanodroplets. The resulting suspension was centrifuged at 10,000 × g for 5 min. After removal of the supernatant, the particles were resuspended in 4 mL of PBS and washed three times using the same centrifugation procedure to remove unincorporated lipids and excess stabilizing components. Following the final washing step, the purified PCNDs were resuspended in 2 mL of PBS, adjusted to a nominal concentration of 1.0 × 10⁹ particles/mL using a hemocytometer, and stored at 4 °C until use.

The hydrodynamic size distribution of the PCNDs was measured by dynamic light scattering (DLS). Surface charge was evaluated by zeta-potential measurements in both PBS and the chlorhexidine-containing treatment medium. Particle morphology and core–shell structure were examined by transmission electron microscopy (TEM), and storage stability was assessed by repeating the DLS measurements after storage at 4 °C for 30 days.

Freshly prepared PCNDs exhibited a mean hydrodynamic diameter of approximately 144 nm. The measured zeta potential was approximately -9.4 mV in PBS and -15.1 mV in the chlorhexidine-containing treatment medium. TEM images demonstrated predominantly spherical nanodroplets with a distinguishable core–shell structure, with most particles distributed within a diameter range of approximately 100–200 nm. After storage at 4 °C for 30 days, the mean hydrodynamic diameter increased slightly to approximately 163 nm. The limited change in particle size and the absence of marked broadening in the size distribution indicated acceptable colloidal stability during the evaluated storage period. A summary of the physicochemical characterization is provided in Table S8. Representative particle-size distribution and zeta-potential results are presented in Figure S1, while the TEM and optical microscopy images of the prepared PCNDs are shown in Figure S2.

**Table S8.** Physicochemical characteristics of the PFP-based phase-change nanodroplet formulation.

| Characteristic | Result |
| --- | --- |
| Phase-change liquid core | Perfluoropentane |
| Interfacial stabilizing layer | DSPC, DSPE-PEG-2000, and Pluronic |
| DSPC amount | 10 mg |
| DSPE-PEG-2000 amount | 4 mg |
| Pluronic amount | 8.7 mg |
| Mean hydrodynamic diameter | Approximately 144 nm |
| Morphological size range | Approximately 100–200 nm |
| Particle morphology | Predominantly spherical particles with a core–shell structure |
| Zeta potential in PBS | Approximately -9.4 mV |
| Zeta potential in chlorhexidine-containing medium | Approximately -15.1 mV |
| Nominal working concentration | 1.0 × 10⁹ particles/mL |
| Storage temperature before experimental use | 4 °C |
| Mean hydrodynamic diameter after storage | Approximately 163 nm |

**Supplementary Figure S1.** Particle-size distribution and zeta-potential characterization of PFP-based phase-change nanodroplets. (a) Representative particle-size distribution of freshly prepared PCNDs, showing a mean hydrodynamic diameter of approximately 144 nm. (b) Zeta potentials of the PCNDs measured at pH 6.3 and 7.4, with values of approximately −9.4 and −15.1 mV, respectively.

**Supplementary Figure S2.** Morphological characterization of PFP-based phase-change nanodroplets. (a) Representative transmission electron microscopy image showing the morphology of the prepared PCNDs. The particles were predominantly spherical and exhibited a distinguishable core–shell structure, with most observed diameters ranging from approximately 100 to 200 nm. (b) Representative optical microscopy image showing the overall dispersion of the prepared PCNDs in suspension.

## 3.2 Technical Specifications of the Ultrasound System

Ultrasound treatment was performed using an ultrasonic therapeutic apparatus (UT1041, Sonic-Stimu Pro; Shenzhen Dongdixin Technology Co., Ltd., Shenzhen, China). The system is equipped with a multi-frequency ultrasound transducer capable of operating at both 1 and 3 MHz.

In the present study, ultrasound frequencies of 1 MHz and 3 MHz were employed. The nominal acoustic intensities were set to 1, 2, and 3 W/cm², with a 50% duty cycle and a treatment duration of 5 min. A flat circular transducer (10 mm diameter) was positioned perpendicular to the specimen surface at a fixed distance of 5 mm. During treatment, tooth specimens were immersed in PBS containing PCNDs at a concentration of 1.0 × 10⁹ particles/mL to ensure consistent acoustic coupling throughout the experiment.

A duty cycle of 50% was used throughout the study because pulsed ultrasound maintains sufficient peak acoustic pressure for acoustic droplet vaporization and cavitation while reducing the time-averaged acoustic energy delivered to the sample, thereby minimizing excessive temperature rise and favoring cavitation-mediated mechanical effects.

## 3.3 Quantitative Analysis of Biofilm Removal

Following image acquisition, microscopic images were subjected to standardized digital image analysis. Images from all experimental groups were analyzed using identical processing parameters. Biofilm-covered and disrupted regions were identified based on image intensity and structural continuity. The biofilm removal rate was calculated as the percentage of disrupted biofilm area relative to the total biofilm-covered area. To minimize operator bias, identical analysis settings were applied to all specimens throughout the study.

## 3.4 Statistical Analysis of Biofilm Removal

To provide a complete statistical description while maintaining the readability of the main manuscript, detailed statistical analyses of the biofilm removal data in Figure 6 are summarized below.

A two-way analysis of variance (ANOVA) was performed with ultrasound frequency (1 and 3 MHz) and ultrasound intensity (1, 2, and 3 W/cm²) as independent variables.

The analysis demonstrated:

- Frequency: F(1,30) = 0.129, P = 0.7215, partial η² = 0.004
- Intensity: F(2,30) = 274.081, P < 0.0001, partial η² = 0.948
- Frequency × Intensity: F(2,30) = 1.281, P = 0.2925, partial η² = 0.079

These results indicate that ultrasound intensity exerted a dominant effect on biofilm removal, whereas neither frequency nor the interaction between frequency and intensity showed statistically significant effects.

# 4 Applicability of the Representative-Bubble Approximation and Reciprocal Bubble–Acoustic-Field Coupling

## 4.1 Scope and rationale of the supplementary analysis

The original Yang–Church–Pitt (YCP) model describes the coupled phase-transition, heat-transfer, and radial-dynamic behavior of a representative ultrasound-activated PCND. The point-scale formulation is intended to identify how incident pressure, frequency, initial droplet radius, surface tension, viscosity, and effective elasticity regulate the local response of a vaporized PCND. It is not intended to reconstruct the complete three-dimensional distribution and collective response of all activated bubbles in the root canal.

Because the experimental suspension contains a population of PCNDs, the response of each vaporized bubble may be influenced by neighboring bubbles. Multi-bubble effects can be described at progressively increasing levels of complexity, ranging from coupled radial equations with secondary radiated pressure to formulations that include propagation delay, translation, secondary Bjerknes forces, multiple scattering, bubble-size distributions, coalescence, fragmentation, and nonlinear wave propagation through a bubble cloud. Complete population-scale descriptions require experimentally resolved post-vaporization bubble-number density, size distribution, activation fraction, spatial distribution, lifetime, phase relationship, and boundary interaction.

These quantities were not directly measured in the present root-canal experiments. Therefore, the purpose of this supplementary analysis was not to construct a complete bubble-cloud model. Instead, a reciprocal two-bubble extension was introduced as a reduced-order applicability test. The analysis addressed two questions: (1) how strongly does a neighboring bubble modify the principal dynamic quantities predicted by the original YCP model; and (2) does this correction alter the main dependence of the representative-bubble response on incident acoustic pressure?

## 4.2 Model parameters and representative separation distances

The ultrasound, PCND, medium, and thermodynamic parameters used in the calculations are reported in Supplementary Table S1. They were selected from the experimental exposure conditions, PCND preparation or characterization, established thermophysical properties of PFP and the surrounding medium, and the original Yang–Church and Pitt formulations. The parameters were not fitted to force agreement between the single- and two-bubble calculations.

The separation distances used in Supplementary Figure S3a were related to the experimental precursor PCND concentration:

$$\begin{aligned} \mathbf{n}\mathbf{=1.0}\boldsymbol{\times}\mathbf{10}^{\mathbf{9}} \text{particles}\text{/}\text{mL}\mathbf{=1.0}\boldsymbol{\times}\mathbf{10}^{\mathbf{15}} \text{m}^{\text{-3}}\boldsymbol{\#}\left( \mathbf{S}\mathbf{21} \right) \end{aligned}$$

Under an equal-cubic-volume approximation, the characteristic center-to-center spacing is:

$$\begin{aligned} \mathbf{d}_{\text{cube}}\mathbf{=}\mathbf{n}^{\mathbf{-1}\mathbf{/}\mathbf{3}}\boldsymbol{=10.0 \mu}\text{m}\boldsymbol{\#}\left( \mathbf{S22} \right) \end{aligned}$$

For a homogeneous three-dimensional Poisson point process, the nearest-neighbor distance has the probability-density function:

$$\begin{aligned} \boldsymbol{f}\left( \boldsymbol{r} \right)\boldsymbol{=4}\boldsymbol{\pi n}\boldsymbol{r}^{\boldsymbol{2}}\boldsymbol{exp}\left( \boldsymbol{-}\frac{\boldsymbol{4}\boldsymbol{\pi n}\boldsymbol{r}^{\boldsymbol{3}}}{\boldsymbol{3}} \right)\boldsymbol{\#}\left( \boldsymbol{S}\boldsymbol{23} \right) \end{aligned}$$

The mode of this distribution is obtained by setting df/dr = 0:

$$\begin{aligned} \boldsymbol{r}_{\text{mode}}\boldsymbol{=}\left( \frac{\boldsymbol{1}}{\boldsymbol{2}\boldsymbol{\pi n}} \right)^{\boldsymbol{1}\boldsymbol{/}\boldsymbol{3}}\boldsymbol{=5.42}\boldsymbol{\mu}\text{m}\boldsymbol{\#}\left( \boldsymbol{S}\boldsymbol{24} \right) \end{aligned}$$

Accordingly, 10 μm represents the equal-cubic-volume characteristic spacing, whereas 5.42 μm represents the most probable nearest-neighbor distance under a spatially random distribution. A separation of 20 μm was included as a weaker-interaction comparison. These values characterize the precursor-number-density scale and should not be interpreted as direct measurements of the post-vaporization bubble distribution. The actual bubble spacing may additionally depend on activation fraction, translation, clustering, coalescence, and geometrical confinement.

**Supplementary Figure S3.** Sensitivity of representative-bubble dynamics to simplified reciprocal bubble–bubble coupling. (a) Normalized radius–time responses (R/R_0_) predicted by the original single-bubble Yang–Church–Pitt (YCP) model and the coupled two-bubble model at center-to-center distances of 20, 10, and 5.42 μm. At the experimental precursor concentration of 1.0 × 10⁹ particles/mL, 10 μm corresponds to the equal-cubic-volume characteristic spacing (n⁻¹/³), whereas 5.42 μm is the mode of the nearest-neighbor distance for a homogeneous three-dimensional Poisson distribution [(1/2πn)¹/³]; 20 μm represents a weaker-interaction comparison. (b) Relative deviation in maximum expansion radius (ΔR_max_) between the coupled and single-bubble models as a function of normalized separation (d/R_max,single_) under incident acoustic pressures of 173, 245, and 300 kPa. (c) Relative deviation in the first-collapse-related time metric (Δt). (d) Relative deviation in the calculated mechanical work (ΔW). The interaction-induced corrections decreased progressively with increasing normalized separation, indicating convergence toward the representative single-bubble response.

## 4.3 Reciprocal two-bubble extension of the YCP model

Two spherical vaporized PCND-derived bubbles were assigned identical initial material and thermodynamic properties. The original YCP phase-transition, thermal, and viscoelastic radial-dynamic equations were retained for each bubble. The incident-only acoustic pressure in the radial equation was replaced by an effective pressure containing both the externally applied pressure and the secondary pressure radiated by the neighboring bubble.

For bubble i:

$$\begin{aligned} \boldsymbol{P}_{\text{eff}\boldsymbol{,i}}\left( \boldsymbol{t} \right)\boldsymbol{=}\boldsymbol{P}_{\text{inc}}\left( \boldsymbol{t} \right)\boldsymbol{+}\boldsymbol{p}_{\text{sc}\boldsymbol{,j}\boldsymbol{\to}\boldsymbol{i}}\left( \boldsymbol{t} \right)\boldsymbol{,i\neq j}\boldsymbol{\#}\left( \boldsymbol{S}\boldsymbol{25} \right) \end{aligned}$$

Under the spherical-radiation approximation, the pressure produced by bubble j at the center of bubble i was calculated as:

$$\begin{aligned} \boldsymbol{p}_{\text{sc}\boldsymbol{,j}\boldsymbol{\to}\boldsymbol{i}}\left( \boldsymbol{t} \right)\boldsymbol{=}\frac{\boldsymbol{\rho}}{\boldsymbol{d}_{\boldsymbol{ij}}}\left[ \boldsymbol{R}_{\boldsymbol{j}}^{\boldsymbol{2}}\left( \boldsymbol{t} \right)\ddot{\boldsymbol{R}_{\boldsymbol{j}}}\left( \boldsymbol{t} \right)\boldsymbol{+2}\boldsymbol{R}_{\boldsymbol{j}}\left( \boldsymbol{t} \right)\dot{\boldsymbol{R}_{\boldsymbol{j}}^{\boldsymbol{2}}}\left( \boldsymbol{t} \right) \right]\boldsymbol{\#}\left( \boldsymbol{S}\boldsymbol{26} \right) \end{aligned}$$

Here, $R_{j}\left( t \right)$, $\dot{R}_{j}\left( t \right)$, and $\ddot{R}_{j}\left( t \right)$ denote the instantaneous radius, wall velocity, and wall acceleration of bubble j, respectively; $\rho$ is the density of the surrounding medium; and $d_{\mathrm{ij}}$ is the center-to-center distance between the two bubbles.

The interaction was implemented reciprocally:

$$\begin{aligned} \boldsymbol{P}_{\text{eff}\boldsymbol{,1}}\left( \boldsymbol{t} \right)\boldsymbol{=}\boldsymbol{P}_{\text{inc}}\left( \boldsymbol{t} \right)\boldsymbol{+}\boldsymbol{p}_{\text{sc}\boldsymbol{,2}\boldsymbol{\to}\boldsymbol{1}}\left( \boldsymbol{t} \right)\boldsymbol{\#}\left( \boldsymbol{S}\boldsymbol{27} \right) \end{aligned}$$

$$\begin{aligned} \boldsymbol{P}_{\text{eff}\boldsymbol{,2}}\left( \boldsymbol{t} \right)\boldsymbol{=}\boldsymbol{P}_{\text{inc}}\left( \boldsymbol{t} \right)\boldsymbol{+}\boldsymbol{p}_{\text{sc}\boldsymbol{,1}\boldsymbol{\to}\boldsymbol{2}}\left( \boldsymbol{t} \right)\boldsymbol{\#}\left( \boldsymbol{S}\boldsymbol{28} \right) \end{aligned}$$

Thus, the external acoustic field drove both bubbles, while each oscillating bubble acted as a secondary acoustic source for the other. The phase-transition and interface-temperature variables were solved concurrently for each bubble, preserving the coupled thermodynamic and mechanical structure of the original YCP model.

The present reduced-order formulation represents the most direct radial acoustic-feedback pathway. It does not include bubble translation, secondary Bjerknes-force-driven migration, coalescence, fragmentation, nonspherical collapse, complete multiple scattering, or realistic root-canal boundary conditions.

## 4.4 Comparison metrics and numerical validity

The original and coupled solutions were compared using the normalized radius–time response, maximum expansion radius, first-collapse-related time, and mechanical work.

The relative maximum-radius deviation was defined as:

$$\begin{aligned} \boldsymbol{\Delta}\boldsymbol{R}_{\boldsymbol{max}}\boldsymbol{=}\frac{\boldsymbol{R}_{\boldsymbol{max,}\text{coupled}}\boldsymbol{-}\boldsymbol{R}_{\boldsymbol{max,}\text{single}}}{\boldsymbol{R}_{\boldsymbol{max,}\text{single}}}\boldsymbol{\times}\boldsymbol{100\%\#}\left( \boldsymbol{S}\boldsymbol{29} \right) \end{aligned}$$

The relative time deviation was defined as:

$$\begin{aligned} \boldsymbol{\Delta}\boldsymbol{t=}\frac{\boldsymbol{t}_{\text{coupled}}\boldsymbol{-}\boldsymbol{t}_{\text{single}}}{\boldsymbol{t}_{\text{single}}}\boldsymbol{\times}\boldsymbol{100\%\#}\left( \boldsymbol{S}\boldsymbol{30} \right) \end{aligned}$$

The relative mechanical-work deviation was defined as:

$$\begin{aligned} \boldsymbol{\Delta}\boldsymbol{W=}\frac{\boldsymbol{W}_{\text{coupled}}\boldsymbol{-}\boldsymbol{W}_{\text{single}}}{\boldsymbol{W}_{\text{single}}}\boldsymbol{\times}\boldsymbol{100\%\#}\left( \boldsymbol{S}\boldsymbol{31} \right) \end{aligned}$$

The center-to-center distance was normalized using the corresponding single-bubble maximum radius:

$$\begin{aligned} \boldsymbol{d}^{\boldsymbol{*}}\boldsymbol{=}\frac{\boldsymbol{d}}{\boldsymbol{R}_{\boldsymbol{max,}\text{single}}}\boldsymbol{\#}\left( \boldsymbol{S}\boldsymbol{32} \right) \end{aligned}$$

This normalization expresses the separation relative to the characteristic maximum bubble size and enables comparison across different incident-pressure conditions.The same droplet, medium, thermal, and acoustic parameters were used for the paired and single-bubble calculations except for the coupling term and the specified separation.

## 4.5 Influence of bubble–bubble interaction on representative-bubble metrics

Supplementary Figure S3(a) compares the original single-bubble radius–time response with the reciprocally coupled responses at center-to-center distances of 20, 10, and 5.42 μm. The 10-μm case represents the equal-cubic-volume characteristic spacing estimated from the experimental precursor concentration. The 5.42-μm case represents the mode of the nearest-neighbor distance under a homogeneous three-dimensional Poisson distribution, whereas 20 μm represents a weaker-interaction condition.

The shortest separation produced the greatest reduction in maximum expansion and the clearest modification of the collapse trajectory. As the center-to-center distance increased, the coupled solution progressively approached the original single-bubble response.

Supplementary Figure S3(b) shows that ΔR_max_ was negative across the investigated conditions, indicating that the reciprocal interaction reduced the maximum expansion relative to the isolated-bubble calculation. The largest reduction was approximately 8% at the smallest normalized separation. The deviation progressively decreased toward zero as d/R_max,single_ increased.

Supplementary Figure S3(c) shows a positive but small change in the first-collapse-related time metric. The largest change was approximately 3%, and it decreased with increasing normalized separation.

Supplementary Figure S3(d) shows that the calculated mechanical work was reduced by approximately 3% or less at the shortest normalized separation and progressively approached the single-bubble value at larger separations. Thus, within the investigated range, the neighboring bubble produced a measurable correction to the absolute local dynamics but did not generate a disproportionately large change in the mechanical-work metric.

The three incident-pressure conditions showed the same distance-dependent convergence pattern. This finding identifies normalized separation as an important variable governing the applicability of the representative-bubble approximation.

## 4.6 Reciprocal acoustic coupling and preservation of the pressure-dependent trend

Supplementary Figure S4(a) illustrates the physical basis of reciprocal acoustic coupling. The incident pressure p_1_ generated by the transducer drives the two bubbles. Bubble 1 then generates the secondary pressure p_sc,1→2_ acting on bubble 2, while bubble 2 generates p_sc,2→1_ acting on bubble 1. The local pressure acting on either bubble is therefore the superposition of the incident and neighboring-bubble-generated pressure components.

Supplementary Figure S4(b) compares the maximum radius predicted by the single-bubble YCP model with the interaction-corrected responses under weak (d/R_max,single_ = 20) and stronger (d/R_max,single_ = 3) interaction conditions.

Increasing the incident pressure from 173 to 300 kPa increased the maximum bubble radius in all three calculations. The weak-interaction result remained close to the original single-bubble prediction, whereas the stronger-interaction condition produced a clearer reduction in absolute maximum radius. Nevertheless, the monotonic pressure dependence and the ordering of the pressure conditions were preserved.

These results demonstrate that reciprocal acoustic interaction should not be ignored conceptually because it produces a measurable quantitative correction. However, under the investigated conditions, the correction does not reverse the central pressure-dependent trend predicted by the representative-bubble YCP model.

**Supplementary Figure S4.** Reciprocal acoustic coupling and its influence on the pressure-dependent representative-bubble response. (a) Schematic illustration of the bidirectional interaction between two neighboring vaporized PCND-derived bubbles. The incident acoustic pressure (p_1_) drives bubble oscillation, while each bubble generates a secondary scattered pressure acting on the neighboring bubble; d denotes the center-to-center separation. (b) Maximum bubble radius predicted by the original single-bubble YCP model and the interaction-corrected models under weak (d/R_max,single_ = 20) and stronger (d/R_max,single_ = 3) interaction conditions. Reciprocal coupling changed the absolute maximum radius, whereas the monotonic increase in bubble radius with incident acoustic pressure was retained.

## 4.7 Interpretation, limitations, and implications

The supplementary results support a layered interpretation of the numerical and experimental findings. At the point scale, the YCP model describes the local phase-transition and radial-dynamic response of a representative vaporized PCND. At the nearest-neighbor scale, the reciprocal two-bubble model quantifies the correction introduced by an adjacent oscillating bubble. At the experimental scale, biofilm-removal and temperature measurements evaluate the treatment-level trend and bulk thermal behavior.

The analysis does not demonstrate that all activated bubbles oscillate independently or that bubble–bubble interaction is universally negligible. It instead shows that the principal incident-pressure dependence used in the mechanistic interpretation remains stable after a simplified reciprocal interaction is introduced.

The complete collective response may additionally depend on activation fraction, bubble-number density, size and phase distributions, translation, secondary Bjerknes forces, clustering, acoustic shielding, multiple scattering, coalescence, fragmentation, nonspherical collapse, and the root-canal and biofilm boundaries. These effects remain outside the scope of the present reduced-order analysis.

Future studies should combine direct measurement of post-vaporization bubble size, concentration, lifetime, and spatial distribution with passive cavitation detection and spatially resolved acoustic-field measurements. Such data would enable the development and validation of fully coupled acoustic-wave–bubble-population models in realistic root-canal geometries. The present analysis provides a useful intermediate step by identifying the principal interaction variables and by demonstrating the level at which the representative-bubble framework remains informative.

# 5 Detailed Protocols for Biofilm Efficacy Assessment

The removal rate mentioned in the main text was obtained by quantitative analysis in MATLAB after microscopic photography in the following ways.

## 5.1 Scanning Electron Microscopy (SEM) Observation

Sample Preparation: After ultrasonic treatment, tooth samples were rinsed 3 times with PBS to remove loosely attached bacteria and nanodroplets. Samples were then fixed in 2.5% glutaraldehyde solution at 4°C for 24 h.

Dehydration: Fixed samples were dehydrated through a graded ethanol series (30%, 50%, 70%, 80%, 90%, 95%, 100%) for 15 min per gradient, followed by critical-point drying with liquid CO₂.

Coating: Dried samples were sputter-coated with a 10-nm thick gold layer to enhance electrical conductivity.

Imaging: Samples were observed using a Hitachi 4800 SEM (acceleration voltage: 10 kV) at ×5000 magnification. Images were captured from 3 random positions on each sample to evaluate biofilm morphology and removal efficiency.

## 5.2 Confocal Laser Scanning Microscopy (CLSM) Analysis

Biofilm Culture on Confocal Dishes: E. faecalis biofilms were cultured on glass-bottom confocal dishes (35 mm) for 7 days to facilitate direct imaging.

Staining: After ultrasonic treatment, dishes were rinsed with PBS and stained with the LIVE/DEAD BacLight Bacterial Viability Kit (Thermo Fisher) for 15 min in the dark. Live bacteria were stained green (SYTO 9), and dead bacteria were stained red (propidium iodide).

Imaging: A Zeiss 710 CLSM was used for imaging with excitation wavelengths of 488 nm (green) and 561 nm (red). Z-stack scanning was performed at 1-μm intervals to generate 3D biofilm images (depth: 60–110 μm). ImageJ software was used to quantify the ratio of live/dead bacteria and biofilm thickness.
